# Specific pathway abundances in the neonatal calf faecal microbiome are associated with susceptibility to *Cryptosporidium parvum* infection: a metagenomic analysis

**DOI:** 10.1186/s42523-023-00265-5

**Published:** 2023-09-12

**Authors:** M. F. Hares, B. E. Griffiths, F. Johnson, C. Nelson, S. Haldenby, C. J. Stewart, J. S. Duncan, G. Oikonomou, J. L. Coombes

**Affiliations:** 1 Infection Biology and Microbiomes, Institute of Infection, Veterinary and Ecological Sciences, University of Liverpool, iC2 Liverpool Science Park, Liverpool, L3 5RF UK; 2 Livestock and One Health, Institute of Infection, Veterinary and Ecological Sciences, University of Liverpool, Leahurst Campus, Neston, Wirral, CH64 7TE UK; 3 Centre of Genomic Research, University of Liverpool, Crown Street, Liverpool, L69 7ZB UK; 4 Translational and Clinical Research Institute, Faculty of Medical Sciences, Newcastle University, Newcastle, NE2 4HH UK; 5 School of Pharmacy and Life Sciences, Robert Gordon University, Garthdee Road, Aberdeen, AB10 7GJ UK

**Keywords:** *Cryptosporidium parvum*, Bovine, Cryptosporidiosis, Faecal microbiome, Metagenome, Pathway abundances, Functional profiling

## Abstract

**Background:**

*Cryptosporidium parvum* is the main cause of calf scour worldwide. With limited therapeutic options and research compared to other *Apicomplexa*, it is important to understand the parasites’ biology and interactions with the host and microbiome in order to develop novel strategies against this infection. The age-dependent nature of symptomatic cryptosporidiosis suggests a link to the undeveloped immune response, the immature intestinal epithelium, and its associated microbiota. This led us to hypothesise that specific features of the early life microbiome could predict calf susceptibility to *C. parvum* infection.

**Results:**

In this study, a single faecal swab sample was collected from each calf within the first week of life in a cohort of 346 animals. All 346 calves were subsequently monitored for clinical signs of cryptosporidiosis, and calves that developed diarrhoea were tested for *Rotavirus*, *Coronavirus*, *E. coli* F5 (K99) and *C. parvum* by lateral flow test (LFT). A retrospective case–control approach was taken whereby a subset of healthy calves (Control group; n = 33) and calves that went on to develop clinical signs of infectious diarrhoea and test positive for *C. parvum* infection via LFT (*Cryptosporidium*-positive group; n = 32) were selected from this cohort, five of which were excluded due to low DNA quality. A metagenomic analysis was conducted on the faecal microbiomes of the control group (n = 30) and the *Cryptosporidium*-positive group (n = 30) prior to infection, to determine features predictive of cryptosporidiosis. Taxonomic analysis showed no significant differences in alpha diversity, beta diversity, and taxa relative abundance between controls and *Cryptosporidium*-positive groups. Analysis of functional potential showed pathways related to isoprenoid precursor, haem and purine biosynthesis were significantly higher in abundance in calves that later tested positive for *C. parvum* (*q* ≤ 0.25). These pathways are either absent or streamlined in the *C. parvum* parasites. Though the de novo production of isoprenoid precursors, haem and purines are absent, *C. parvum* has been shown to encode enzymes that catalyse the downstream reactions of these pathway metabolites, indicating that *C. parvum* may scavenge those products from an external source.

**Conclusions:**

The host has previously been put forward as the source of essential metabolites, but our study suggests that *C. parvum* may also be able to harness specific metabolic pathways of the microbiota in order to survive and replicate. This finding is important as components of these microbial pathways could be exploited as potential therapeutic targets for the prevention or mitigation of cryptosporidiosis in bovine neonates.

**Supplementary Information:**

The online version contains supplementary material available at 10.1186/s42523-023-00265-5.

## Background

*Cryptosporidium parvum* is an apicomplexan, protozoan parasite that invades the small intestinal epithelium of neonatal calves. It causes an acute diarrhoeal disease known as cryptosporidiosis, which is characterised by watery diarrhoea, dehydration, weight loss and even death in severe cases. Cryptosporidiosis leads to approximately 37% of all diarrhoea events and 20% of co-infections in calves in the UK, culminating in production losses of approximately £130 per calf affected and poorer overall animal welfare [1, 2]. Consequently, it is a serious veterinary issue which requires effective therapies to combat infection. With no vaccine available against bovine cryptosporidiosis at this time, the current therapeutic options in cattle are limited to the antibiotic, paromomycin, and the anti-cryptosporidial, FDA-approved drug, halofuginone, which is believed to target the merozoite and sporozoite stages [3]. Unfortunately, both halofuginone and paromomycin have been found to have variable efficacy against cryptosporidiosis in calves. While the cryptosporidiostatic effect can both reduce oocyst shedding and severity of diarrhoea, these treatments are not lethal to *Cryptosporidium* and oocyst shedding and diarrhoea will often commence on drug withdrawal [4–10]. In addition, halofuginone has high toxicity at twice the recommended dose, leading to adverse side effects, therefore calves must be weighed in order to administer an effective, non-lethal dose [3, 11–13]. In light of this, the development of new effective therapies against cryptosporidiosis in calves is crucial, not only from an animal welfare point of view but also from an economic perspective.

In order to develop effective therapies against *C. parvum* in calves, an understanding of how the parasite interacts with the gut environment is important to ascertain the microbes and their metabolites that are associated with health or infection. The gut microbiome plays a significant role in bovine intestinal homeostasis by the production of metabolites that support intestinal epithelial processes to regulate mucosal barrier function and immune responses [14, 15]. In addition, the gut microbiome in healthy animals is protective against infection as it reduces the risk of pathogenic colonisation by competitive exclusion [16]. Disruption of the microbiome by inflammation, changes in diet, antibiotics, probiotics and stress increases the risk of cryptosporidiosis [17–21]. In turn, *Cryptosporidium* has been shown to manipulate the host microbiome [22–24]. Therefore, the causality of the changes in the gut microbiome observed during *C. parvum* infection are ambiguous.

There are some studies that directly sequence the microbiome of the gastrointestinal tract (GIT) of healthy neonatal calves. These studies show how the composition of the microbiome changes along different sections of the GIT during the preweaning stage [25, 26]. This is valuable data in relation to *Cryptosporidium* infection as they report on the microbiome of the ileum; the location of parasite invasion, during the life stage when calves are most vulnerable to *C. parvum* infection. Although there are differences between the faecal and small intestinal microbiomes, faecal microbiome studies are useful as they allow for longitudinal study design that follows the same animal for the study duration, a minimally invasive approach, and remove the requirement to cull large numbers of animals for the collection of tissue and digesta samples.

There is a wealth of data that examines the calf faecal microbiome in relation to health and diarrhoeal disease [27–38]. The general consensus is that species diversity increases over time between birth and weaning and the predominant phyla present during the pre-weaning phase are *Firmicutes*, *Bacteroidetes*, *Actinobacteria,* and *Proteobacteria* [28–31, 33–37]. Furthermore, calf diarrhoea is often associated with lower diversity in the faecal microbiome when compared to healthy controls [28–32, 34–38]. As for specific taxa that are associated with health or diarrhoeal disease, there are some conflicting reports, which is likely due to the differences in the farm location, farm management practices, study design and the use of different databases in sequence alignment. For example, species of the genus *Lactobacillus* have long been associated with calf gut health and have been used as a probiotic to ameliorate signs of diarrhoeal disease in calves [29, 39, 40]. However, this taxon has also been found to be enriched in the faecal microbiomes of calves suffering from diarrhoea [31, 37]. This shows how complex the interactions between the microbiome and gastrointestinal disease are and that there is no straight-forward solution in the development of probiotic therapies. Even with this study variation, some taxa have been exclusively associated with either calf health or diarrhoea in various articles. *Faecalibacterium*, *Barnesiella* and *Bifidobacterium* have all been shown to be significantly enriched in the faecal microbiomes of healthy calves and to reduce the incidence of calf diarrhoea in multiple studies [28–32, 36, 37]. Conversely, various reports agree that *Enterobacteriaceae* and *Fusobacterium* are commonly enriched in the faecal microbiomes of calves that experience diarrhoea [28, 29, 31, 34, 37, 38]. Whether the increased abundance of these taxa is the cause of the diarrhoea or is caused by the diarrhoea itself is unclear and requires further research to ascertain the causality of this trend.

On the other hand, only a limited number of studies exist that focus specifically on the calf faecal microbiome in relation to cryptosporidiosis [41–44]. Several studies have shown that there is a higher abundance of *Fusobacterium* in the faecal microbiome of calves with *C. parvum* infection compared to healthy controls [41, 44]. Yet, as previously cited, this trend is also described in studies that focus on calf diarrhoea in general, so the causality of this association has yet to be determined. Unfortunately, no studies to date have identified specific features of the microbiome prior to infection that may predict calf susceptibility to the development of cryptosporidiosis. Microbiome studies of this nature would be useful for the selection of potential candidates for microbial metabolic inhibitors, pre/pro/post-biotics or alternate therapies such as faecal microbiota transplantation (FMT) [45, 46]. Our study aims to address the knowledge gap.

We hypothesised that specific features of the faecal microbiota of calves, prior to infection, could predict calf susceptibility to cryptosporidiosis. In a retrospective case–control study, we conducted a metagenomic analysis of faecal samples collected from calves during the first week of life (n = 60). The aim of the study was to determine any pre-disposing taxonomic and/or functional characteristics of the microbiome that are associated with susceptibility to cryptosporidiosis in neonatal calves.

## Methods

The study was conducted following ethical approval by the University of Liverpool Research Ethics Committee (VREC927) and procedures regulated by the Animals (Scientific Procedures) Act were conducted under a UK Home Office License (P191F589B).

### Animals

346 female Holstein dairy calves were enrolled on this study from three farms (Farm 1, 2, and 3) based in North Wales and Cheshire, UK. Calves that had received routine antibiotic and/or anti-cryptosporidial prophylactic treatment were included as this is common practice on UK farms. All calves received a similar dietary regime of cow colostrum in the first 24 h of life, followed by milk replacer, and were then weaned onto a standard cereal and hay-based diet. The breed and farm management of the sample population of calves on all farms was considered by the veterinary team as representative of the UK dairy calf population. All of the calves were monitored throughout the study by body condition score (BCS), and the Wisconsin Scoring System, as well as a scoring system developed by the sample collector to determine the health status of the calves [47, 48]. In addition, blood serum total protein was measured within 7 days of birth and thoracic ultrasonography was used to identify respiratory disease post-weaning. All calves included in the study displayed no clinical signs of cryptosporidiosis in the first week of life sampling period. The study design is presented in Additional file 1: Fig. S1.

### Sample collection

One faecal sample was collected from each of the 346 ≤ 1-week-old calves by rectal swab (Sterilin Regular Nylon Flocked Swabs 552C, Scientific Laboratory Supplies), prior to the development of any clinical signs of cryptosporidiosis, and stored on dry ice immediately after the collection. Samples were transferred to − 80 °C within a few hours from the collection and stored until DNA extraction. The health monitoring conducted by experienced veterinary clinicians over the course of the study included a faecal score which was used to determine if a diarrhoea event had occurred [48]. A diarrhoea event was defined as any faecal score of two or more which is described as "loose but enough consistency to remain on bedding" to "watery stool that sifts through bedding". Calves that exhibited a diarrhoea event were tested for infectious agents using a lateral flow test (LFT) (MSD Rainbow Calf Scour Diagnostic Faecal Test, Farmacy) designed to detect the main causes of infectious diarrhoea: *Rotavirus*, *Coronavirus*, *E. coli* F5 (K99) and *Cryptosporidium parvum*. Once an appropriate number of the calves tested positive for *C. parvum* infection following week 1 sampling (n = 32), healthy control calves were selected from the remaining sampled cohort (n = 33). *Cryptosporidium*-positive calves were selected on the basis that they showed clinical signs of diarrhoea and received a positive LFT for *Cryptosporidium parvum* after week 1 sampling. Two of the *Cryptosporidium*-positive calves included in the study also tested positive for *Rotavirus* on the LFT. Healthy control calves were selected on the basis that they showed no clinical signs of diarrhoeal disease during the study period, though calves with mild respiratory disease signs or that had received routine prophylaxis (Diatrim, Synulox and Halocur) were permitted to be included in the study. The control group was matched to the *Cryptosporidium*-positive group by age, sex, farm, and breed and as closely matched for date and type of prophylactic treatment as possible. From here onwards, selected calves that did not experience a diarrhoea event will be referred to as the control group (n = 33) and calves that experienced a diarrhoea event and received a positive test result for *Cryptosporidium* after week 1 sampling will be referred to as the *Cryptosporidium*-positive group (n = 32).

### Sample preparation

Faecal swab samples were placed directly into bead beating tubes provided in the DNA extraction kit (DNeasy PowerLyzer PowerSoil Kit, QIAGEN). Excess plastic applicator was removed using scissors, sterilised with 100% ethanol and a Bunsen burner between samples, to allow swabs to fit into the tubes. DNA extraction was performed on all samples following the manufacturers protocol with the following adjustments; 500 µL of Powerbead solution was added to each tube along with 60 µL of solution C1. Swabs were bead beaten for 15 min in a tube adaptor on the Vortex Genie 2 at 7.5 speed. C2 and C3 were mixed 1:1 and 300 µL of this solution was added to the sample supernatant and placed at 4 °C for 5 min. 50 µL of C6 Elution Buffer was added to the spin column membrane and incubated at room temperature for 5 min to elute the gDNA. Negative extraction controls were provided in the form of empty bead beating tubes and were processed alongside the samples.

DNA was quantified using the Nanodrop and Qubit 3.0 to determine DNA concentration. DNA quality was also determined using the Nanodrop and gel electrophoresis using a 1 Kb ladder. Samples with gDNA quality and quantity that did not meet the Centre for Genomic Research (CGR, University of Liverpool) QC requirements (All samples required to contain 1–500 ng gDNA in ≤ 5 μL; 260:280/260:230 ratio ≥ 1.80) were excluded from the study (n = 5), resulting in a final total of 60 control (n = 30) and *Cryptosporidium*-positive (n = 30) samples as well as three negative extraction controls.

### Shotgun metagenomic sequencing

60 gDNA samples and three negative extraction control samples underwent shotgun metagenomic sequencing and analysis at the CGR, University of Liverpool. The Illumina fragment library was prepared from the gDNA samples using the Illumina NEBNext Ultra II FS kit on the Mosquito platform using the 1/10 reduced volume protocol. 50 ng of DNA was used as input material where available, followed by size selection of Adaptor-ligated DNA. Following 8 cycles of amplification, the libraries were purified using Ampure XP beads. These final libraries were pooled and the quantity and quality of the pool was assessed by Qubit and the Bioanalyzer and later by qPCR using the KAPA Illumina Library Quantification Kit (Roche) on a LightCycler^®^ LC480II (Roche) according to manufacturer's instructions. After calculation of the molarity using qPCR data, template DNA was diluted to 300 pM and denatured for 8 min at room temperature using freshly diluted 0.2 N sodium hydroxide (NaOH) and the reaction was subsequently terminated by the addition of 400 mM TrisCl pH = 8. To improve sequencing quality control, 1% PhiX was spiked-in. The libraries were sequenced on the Illumina^®^ NovaSeq 6000 platform (Illumina^®^, San Diego, USA) following the XP workflow on two lanes of an S4 flow cell, generating 2 × 150 bp paired-end reads. See BioProject: PRJNA935534 to access raw sequence data.

### Data processing

Initial processing and quality assessment of the sequence data was performed. Briefly, base calling and de-multiplexing of indexed reads was performed by CASAVA version 1.8.2 (Illumina). The resulting raw fastq files were trimmed to remove Illumina adapter sequences using Cutadapt version 1.2.1 [49]. The reads were further trimmed to remove low quality bases, using Sickle version 1.2 with a minimum window quality score of 20 [50]. After trimming, reads shorter than 20 bp were removed. Statistics for the total number of reads obtained for each sample and the distribution of trimmed read lengths for the forward (R1), reverse (R2) and singlet (R0) reads were generated using fastq-stats from EAUtils (Additional file 1: Figs. S2 and S3) [51].

Prior to analysis, host reads were removed from all samples by aligning reads to the *Bos taurus* and *Homo sapiens* combined reference genomes, using the short-read alignment tool, Bowtie2 [52]. The resulting alignment file was processed to extract and retain read pairs where neither read aligned to the host genome, using custom scripts (Additional file 2). The percentage of retained reads for each sample are shown in Additional file 1: Table S1.

Samples underwent taxonomic profiling whereby Kraken2 was used to assign a taxonomic ID to each sequence read and Bracken was used to convert the raw counts into predicted relative abundances for each taxon [53, 54]. Bracken relative abundance tables were parsed by taxonomic rank from species up to phylum level using a custom script (Additional file 3). The species relative abundance tables were filtered to 0.1% abundance in at least one sample to remove low abundance species for use in downstream analysis (Additional file 4).

Prior to functional profiling, read pairs from each sample were analysed to detect overlaps and merged accordingly using PEAR [55]. The samples underwent functional profiling using a MetaPhlAn2 generated relative abundance table (Additional file 5) and HUMAnN3 to produce gene family and MetaCyc pathway relative abundances. The gene family relative abundances were converted to GO then GO-Slim term abundances (biological processes, molecular functions, and cellular components) [56–58]. Following processing with HUMAnN3, pathway abundances and GO-Slim terms were renormalised as counts per million reads (CPM).

### Statistical analysis

Diversity was measured and plotted using R version 4.2.2 and R packages: tidyverse 1.3.2, vegan 2.6.2, ape 5.6.2, ggpubr 0.4.0, ggsignif 0.6.4, ggtext 0.1.2, glue 1.6.2, and scales 1.2.1 [59–67]. Comparisons of diversity were made between the control and *Cryptosporidium*-positive groups as well as between the sample collection days by grouping the calves into the first half of the week (Day 1–3) or the latter half of the week (Day 4–7). The alpha diversity of samples was measured using species richness and the Shannon index. Normality tests showed that richness data was normal and Shannon diversity data was not normally distributed. The unpaired T-test was used to determine significant differences in species richness between groups. The unpaired Wilcoxon test was applied to determine significant differences in Shannon diversity between groups. Beta diversity of samples was measured using Bray–Curtis PCoA ordination. A PERMANOVA was used to ascertain whether there was a significant distance between centroids.

Taxa relative abundance stacked bar charts were plotted in R version 4.2.2 and R packages: tidyverse 1.3.2, vegan 2.6.2, RColorBrewer 1.1–3, egg 0.4.5, ggtext 0.1.2, and markdown 1.4 [62, 63, 65, 67–70]. The Multivariate Association with Linear models 2 (MaAsLin2) package version 1.8.0 was used to conduct statistical analysis of HUMAnN3, Bracken and MetaPhlAn2 relative abundance outputs. Comparisons between the control and *Cryptosporidium*-positive groups were performed to reveal any significant taxa or functional data, whilst correcting for confounding variables [71]. Confounding variables included farm (Farm 1, 2, and 3), antibiotic/anti-cryptosporidial treatment (Diatrim, Synulox, and Halocur) and sampling day within the first week of life (Day 1–7). These confounding variables were all included as fixed effects in the MaAsLin2 analysis. Parameters were kept the same for HUMAnN3, Bracken and MetaPhlAn2 data. The minimum abundance was set to 0.0001 and minimum prevalence was set to 0.1. *p*-values were adjusted by MaAsLin2 for multiple comparisons using Benjamini–Hochberg procedure (False Discovery Rate). The *q*-value cut-off was kept at the default value of 0.25 for taxonomic and functional profiling. Datasets were normalised by Total Sum Scaling (TSS) and the transformation parameter (enables logarithmic/arcsine square root functions to be applied to dataset) was set to “NONE”. Significant results (*q* ≤ 0.25) from the MaAsLin2 analysis were visualised using GraphPad Prism 9.3.1 [72]. Study metadata and R analysis code are presented in Additional files 6 and 7, respectively.

## Results

### Microbial diversity does not predict susceptibility to *C. parvum* infection

**Fig. 1:**
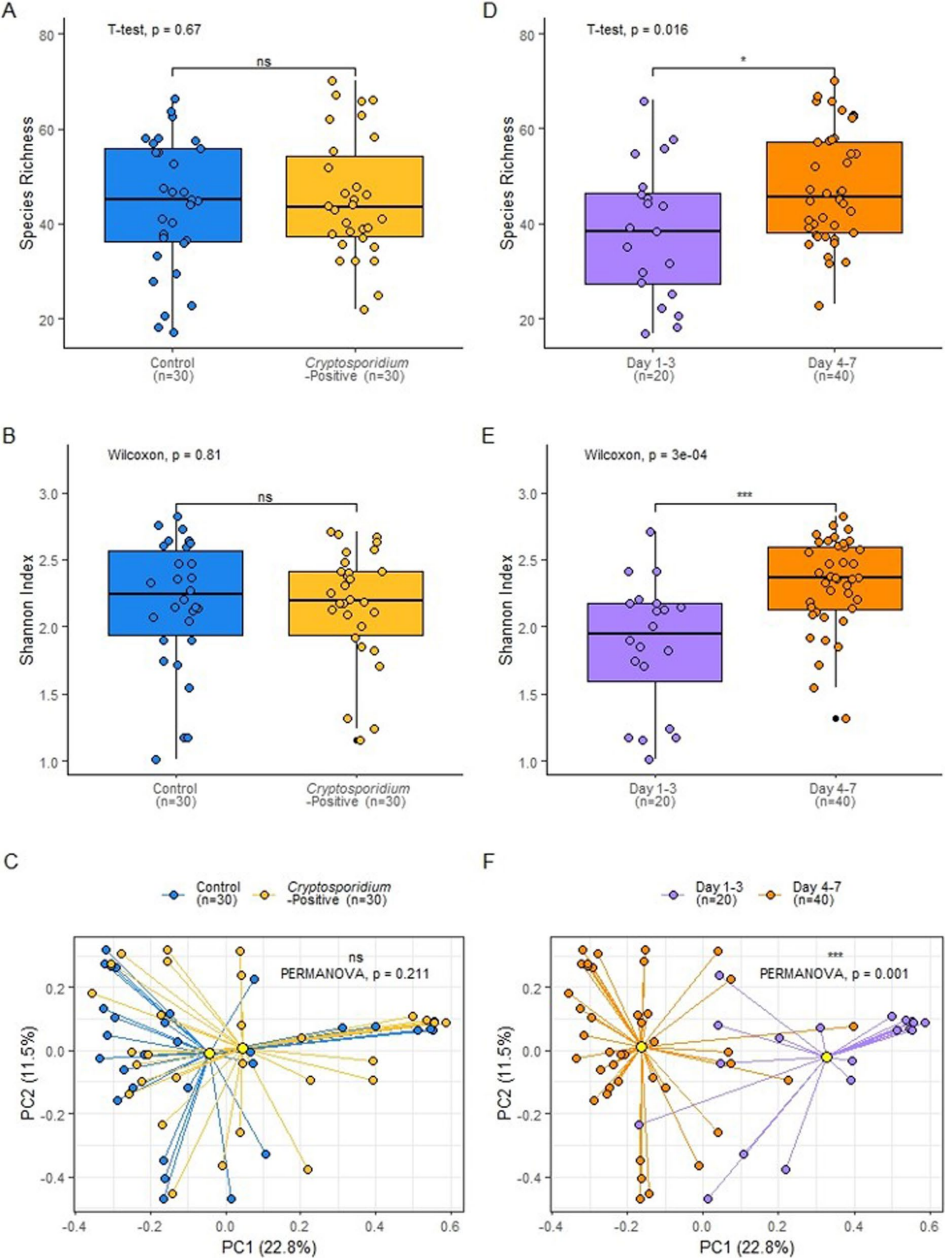
Alpha and beta diversity of control versus *Cryptosporidium-*positive and Day 1–3 versus Day 4–7 sampling groups. **A** Species richness of control (n = 30) and *Cryptosporidium*-positive (n = 30) groups; T-test, *p* = 0.67. **B** Shannon index of control (n = 30) and *Cryptosporidium-*positive (n = 30) groups; Wilcoxon, *p* = 0.81. **C** Bray Curtis PCoA ordination plot of the control (n = 30) and *Cryptosporidium-*positive (n = 30) groups; PERMANOVA, *p* = 0.21. **D** Species richness of calves sampled on Day 1–3 (n = 20) versus Day 4–7 (n = 40); T-test, *p* = 0.016. **E** Shannon index of calves sampled on Day 1–3 (n = 20) versus Day 4–7 (n = 40); Wilcoxon, *p* = 0.0003. **F** Bray Curtis PCoA ordination plot of calves sampled on Day 1–3 (n = 20) versus Day 4–7 (n = 40); PERMANOVA, *p* = 0.001

DNA extracted from faecal samples collected from 60 ≤ 1-week-old calves prior to the onset of *C. parvum* infection underwent shotgun metagenomic sequencing, processing, and taxonomic and functional profiling. Samples were grouped by calves that remained healthy for the duration of the study (Control group; n = 30) and calves that displayed clinical signs and tested positive for *Cryptosporidium* *parvum* infection following sampling (*Cryptosporidium*-positive group; n = 30). A metagenomic analysis was performed to compare various aspects of the microbiomes of control and *Cryptosporidium*-positive groups, to determine features associated with susceptibility to infection. Taxonomic profiling down to species level provided species relative abundance tables that were used to measure alpha and beta diversity of the calf faecal microbiome to determine their impact, if any, on susceptibility to bovine cryptosporidiosis. The early microbial diversity between calves was extremely varied. Control and *Cryptosporidium*-positive groups showed no significant differences in species richness (T-test, *p* = 0.67), Shannon diversity (Wilcoxon, *p* = 0.81) or beta diversity (PERMANOVA, *p* = 0.21; Fig. 1A–C). However, calves sampled on Day 1–3 versus Day 4–7 had a significant difference in species richness (T-test, *p* = 0.016), Shannon diversity (Wilcoxon, *p* = 0.0003) and beta diversity (PERMANOVA, *p* = 0.001; Fig. 1D–F). Calves sampled on Day 1–3 exhibited significantly lower alpha diversity compared to calves sampled on Day 4–7. Calves sampled on Day 1–3 and Day 4–7 showed significant dissimilarity in the Bray–Curtis PCoA. This could be attributed to the rapid diversification of the microbiome in the first week of life observed in the existing literature [73, 74].

### Taxa abundance does not predict susceptibility to *C. parvum* infection

**Fig. 2:**
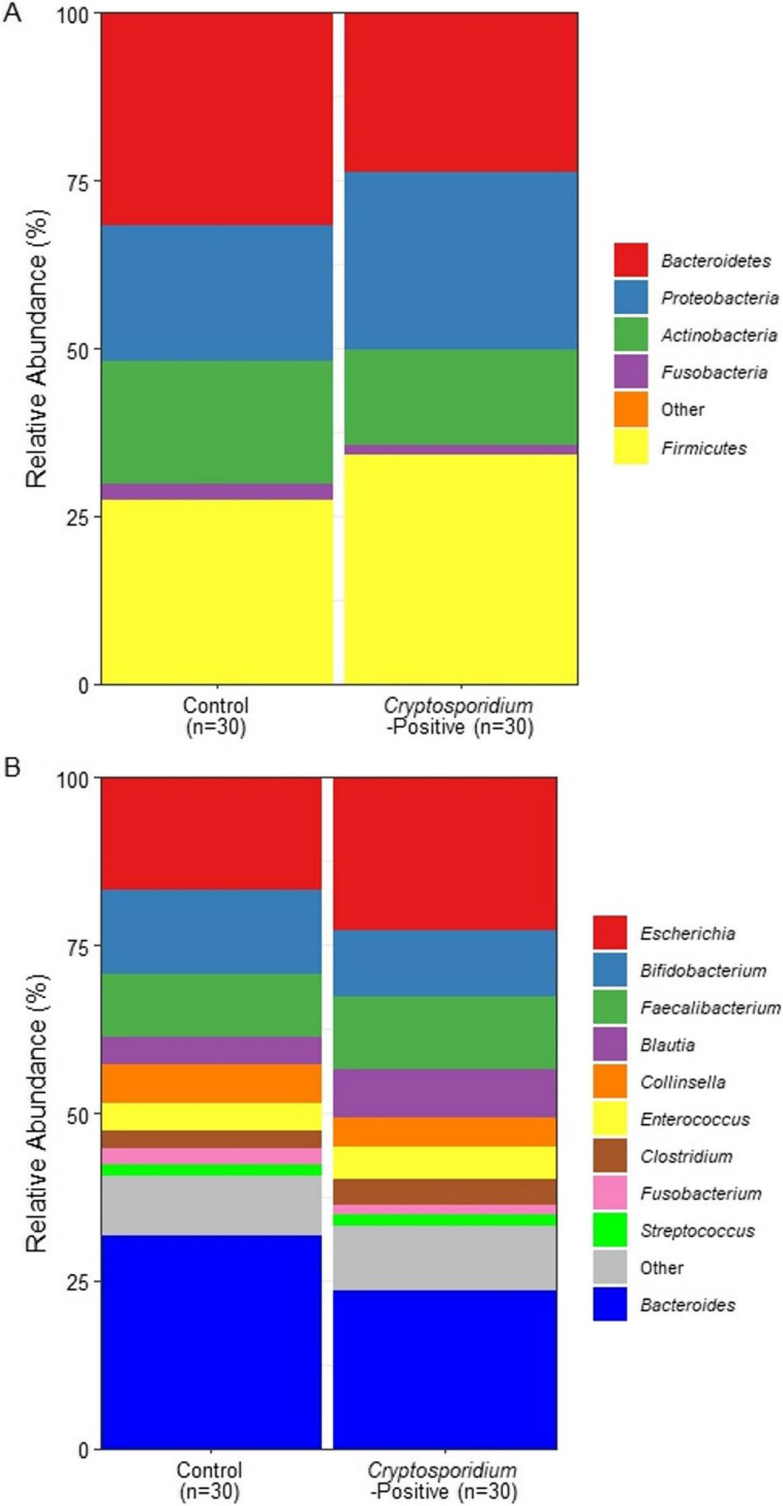
Mean relative abundance of the microbial composition among the control and *Cryptosporidium*-positive groups. **A** Phyla relative abundance (≥ 1%) in the control group (n = 30) versus the *Cryptosporidium*-positive group (n = 30). **B** Genera relative abundance (≥ 1%) in the control group (n = 30) versus the *Cryptosporidium*-positive group (n = 30)

Species relative abundance tables generated by Kraken2/Bracken were parsed by taxonomic rank from species up to phylum level using a custom script and used to compare the relative abundance of different taxa at different taxonomic levels between the control and *Cryptosporidium*-positive groups. The predominant phyla (≥ 1% relative abundance) present in all samples were *Firmicutes* (30.8%), *Bacteroidetes* (27.7%), *Proteobacteria* (23.4%), *Actinobacteria* (16.1%), and *Fusobacteria* (1.96%). The control group had higher relative abundances of *Bacteroidetes* (31.8% vs 23.7%) and *Actinobacteria* (18.2% vs 14.1%), and lower relative abundances of *Firmicutes* (27.4% vs 34.1%) and *Proteobacteria* (20.2% vs 26.6%) compared to the *Cryptosporidium*-positive group (Fig. 2A). At the genus level, the control group had higher relative abundances of *Bacteroides* (31.6% vs 23.5%), *Bifidobacterium* (12.5% vs 9.88%) and *Fusobacterium* (2.44% vs 1.58%), and lower relative abundances of *Escherichia* (16.7% vs 22.9%), *Faecalibacterium* (9.49% vs 10.8%), and *Blautia* (4.00% vs 7.10%) compared to the *Cryptosporidium*-positive group (Fig. 2B). However, these differences did not reach statistical significance in the MaAsLin2 analysis (Additional files 8 and 9).

Two taxonomic profiling tools were used to determine the composition of the calf microbiome; Bracken and MetaPhlAn2. No *Cryptosporidium* sequences were detected in any of the samples in the Kraken2/Bracken raw output, but a very low abundance of *Cryptosporidium hominis* and *Cryptosporidium parvum* sequences (0.003% and 0.004% relative abundance respectively) were found in the MetaPhlAn2 data in one control sample. When the Bracken species relative abundance data was analysed using MaAsLin2, *Veillonella rodentium* was found to be significantly less abundant in the control group compared to the *Cryptosporidium*-positive group (*q* = 0.13; Additional file 1: Fig. S6A; Additional file 10). However, the only non-zero relative abundance samples for this dataset were one in the control group versus eight in the *Cryptosporidium*-positive group. MetaPhlAn2 was run as part of the functional profiling pipeline and the  species relative abundance data was also run through MaAsLin2. The relative abundance of *Veillonella *sp. CAG 933 was found to be significantly lower in the control group compared to the *Cryptosporidium-*positive group in the MetaPhlAn2 data (*q* = 0.19; Additional file 1: Fig. S6B; Additional file 11). The non-zero samples for this species were 13 in the control group and 10 in the *Cryptosporidium*-positive group. Both Bracken and MetaPhlAn2 data have comparable results as the same genus was found to be significant for both datasets. Otherwise, no significant differences in taxa were found in Bracken or MetaPhlAn2 relative abundance data between control and *Cryptosporidium*-positive groups, when adjusting for potential confounding variables using MaAsLin2 (Additional files 10 and 11). This implies that no single taxon was strongly associated with susceptibility to *C. parvum* infection.

### Specific pathway abundances may predict susceptibility to *C. parvum* infection

**Fig. 3:**
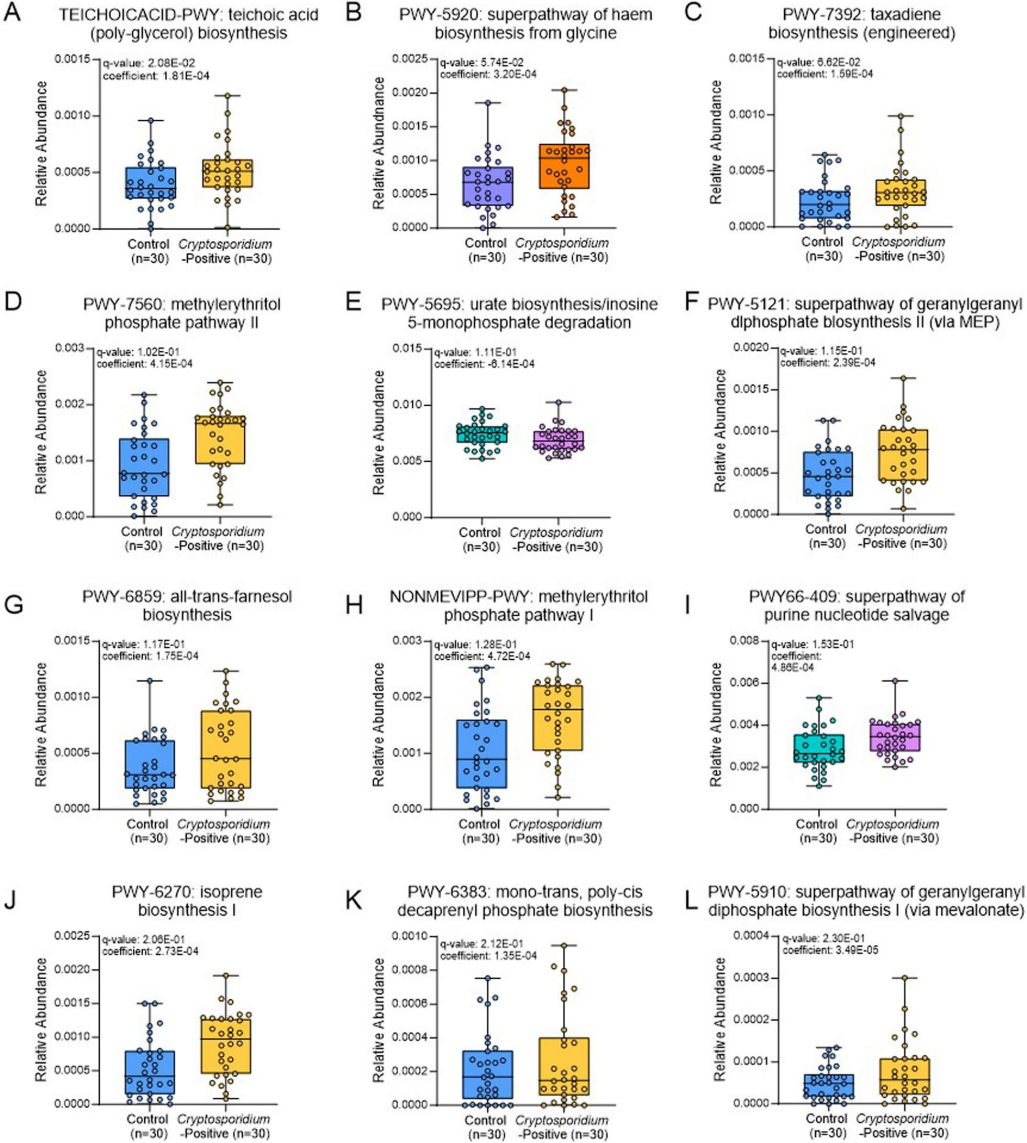
Significant TSS normalised destratified pathway relative abundances of the control and *Cryptosporidium*-positive groups. **A** TEICHOICACID-PWY: teichoic acid (poly-glycerol) biosynthesis; *q* = 0.021. **B** PWY-5920: superpathway of haem biosynthesis from glycine; *q* = 0.057. **C** PWY-7392: taxadiene biosynthesis (engineered); *q* = 0.066. **D** PWY-7560: methylerythritol phosphate pathway II; *q* = 0.10. **E** PWY-5695: urate biosynthesis/inosine 5-monophosphate degradation; *q* = 0.11. **F** PWY-5121: superpathway of geranylgeranyl diphosphate biosynthesis II (via MEP); *q* = 0.12. **G** PWY-6859: all-trans-farnesol biosynthesis; *q* = 0.12. **H** NONMEVIPP-PWY: methylerythritol phosphate pathway I; *q* = 0.13. **I** PWY66-409: superpathway of purine nucleotide salvage; *q* = 0.15. **J** PWY-6270: isoprene biosynthesis I; *q* = 0.21. **K** PWY-6383: mono-trans, poly-cis decaprenyl phosphate biosynthesis; *q* = 0.21. **L** PWY-5910: superpathway of geranylgeranyl diphosphate biosynthesis I (via mevalonate); *q* = 0.23

Though there were no significant differences in the microbiome diversity or taxa prior to the onset of *C. parvum* infection, the metagenomic analysis also allows for the determination of the functional potential of specific taxa. Functional profiling was performed using HUMAnN3 with MetaPhlAn2 and significant differences between the control and *Cryptosporidium*-positive groups in the resulting destratified (taxa contributions removed to show only community abundances) functional relative abundance tables were determined (Additional file 12). The multivariate analysis showed that 12 MetaCyc pathway relative abundances between the control and *Cryptosporidium*-positive groups were significantly different (*q* ≤ 0.25; Fig. 3; Table 1; Additional file 1: Fig. S7; Additional file 13). The majority of these pathways were related to the methylerythritol phosphate (MEP) pathway for the biosynthesis of isoprenoid precursors. Others were related to purine salvage and degradation, and haem biosynthesis.

**Table 1:** Significant pathway relative abundances in the control group (n = 30) versus the *Cryptosporidium*-positive group (n = 30).

MetaCyc pathway	Biological function	Coefficient value*	Association with *C. parvum*	Taxa contributing to pathway	*p*-value	*q*-value (adjusted *p*-value)
TEICHOICACID-PWY: teichoic acid (poly-glycerol) biosynthesis	Interacts with isoprenoids and forms Gram-positive bacterial cell wall	1.81E−04	Positive	*Escherichia coli, Staphylococcus condimenti,* unclassified	0.002293	0.020765
PWY-5920: superpathway of haem biosynthesis from glycine	Cytochromes use haem as a cofactor	3.20E−04	Positive	*Escherichia coli,* unclassified	0.007606	0.057446
PWY-7392: taxadiene biosynthesis (engineered)	Intermediate of isoprenoid biosynthesis converts to taxol precursor	1.59E−04	Positive	Unclassified	0.009125	0.066171
PWY-7560: methylerythritol phosphate pathway II	Isoprenoid precursor biosynthesis	4.15E−04	Positive	*Escherichia coli, Klebsiella pneumonia, Proteus mirabilis,* unclassified	0.014846	0.102318
PWY-5695: urate biosynthesis/inosine 5-monophosphate degradation	Purine nucleotide degradation	− 6.14E−04	Negative	*Alistipes, Allisonella, Anaerostipes, Anaerotignum, Bacteroides, Bibersteinia, Bilophila, Ruminococcus, Catenibacterium, Citrobacter, Clostridioides, Clostridium, Desulfovibrio, Enterobacter, Enterococcus, Clostridium, Escherichia, Flavonifractor, Fusicatenibacter, Fusobacterium, Gallibacterium, Hafnia, Intestinibacter, Klebsiella, Kluyvera, Kocuria, Lactococcus, Mannheimia, Megamonas, Megasphaera, Morganella, Parabacteroides, Pasteurella, Prevotella, Proteus, Providencia, Pseudoflavonifractor, Raoultella, Staphylococcus, Streptococcus, Terrisporobacter, Vagococcus, Veillonella, and* unclassified spp. (Species are grouped by genera here for brevity)	0.016182	0.110565
PWY-5121: superpathway of geranylgeranyl diphosphate biosynthesis II (via MEP)	Isoprenoid precursor biosynthesis	2.39E−04	Positive	*Escherichia coli, Klebsiella pneumonia, Proteus mirabilis,* unclassified	0.017217	0.115303
PWY-6859: all-trans-farnesol biosynthesis	Isoprenoid precursor biosynthesis	1.75E−04	Positive	*Citrobacter portucalensis, Citrobacter youngae, Enterobacter cloacae complex, Escherichia coli, Escherichia fergusonii, Klebsiella oxytoca, Klebsiella pneumonia, Morganella morganii, Proteus mirabilis, Proteus vulgaris, Providencia stuartii,* unclassified	0.01747	0.116663
NONMEVIPP-PWY: methylerythritol phosphate pathway I	Isoprenoid precursor biosynthesis	4.72E−04	Positive	*Escherichia coli, Klebsiella pneumonia, Proteus mirabilis,* unclassified	0.019814	0.128331
PWY66-409: superpathway of purine nucleotide salvage	Purine nucleotide salvage	4.86E−04	Positive	*Clostridium butyricum, Clostridium perfringens, Clostridium sp 7 2 43FAA, Enterobacter cloacae complex, Enterococcus faecalis, Enterococcus faecium, Escherichia coli, Klebsiella oxytoca, Klebsiella pneumonia, Proteus mirabilis, Providencia stuartii, Streptococcus equinus, Streptococcus gallolyticus, Streptococcus pasteurianus,* unclassified	0.023986	0.15325
PWY-6270: isoprene biosynthesis I	Isoprenoid precursor biosynthesis	2.73E−04	Positive	*Escherichia coli, Klebsiella pneumonia, Proteus mirabilis,* unclassified	0.034367	0.206205
PWY-6383: mono-trans, poly-cis decaprenyl phosphate biosynthesis	Isoprenoid precursor biosynthesis	1.35E−04	Positive	Unclassified	0.035707	0.212223
PWY-5910: superpathway of geranylgeranyl diphosphate biosynthesis I (via mevalonate)	Isoprenoid precursor biosynthesis	3.49E−05	Positive	Unclassified	0.039785	0.229955

*The coefficient value is indicative of the effect size

### Isoprenoid precursor related pathways are associated with susceptibility to *C. parvum* infection

Several isoprenoid precursor-associated MetaCyc pathway relative abundances were significantly lower in the control group compared to the *Cryptosporidium*-positive group (*q* ≤ 0.25; Fig. 3A, C-D, F–H, J-L; Table 1). The HUMAnN3 stratified data (attributes taxa contributions to the functional abundances) further shows that *Escherichia coli, Klebsiella pneumoniae* and *Proteus mirabilis* species were responsible for the relative abundance of MEP-related pathways in the control group and the *Cryptosporidium*-positive group, though the remainder of the species involved in these pathways were unclassified (Table 1; Additional file 14). TEICHOICACID-PWY, PWY-7392, PWY-7560, PWY-5121, PWY-6859, NONMEVIPP-PWY, PWY-6270, and PWY-6383 were the significant pathways related to the MEP pathway (Additional file 1: Fig. S8) and PWY-5910 was linked to the mevalonate (MVA) pathway.

In addition, the data shows that *Escherichia coli*, *Staphylococcus condimenti* and unclassified species were responsible for the relative abundance of the teichoic acid (poly-glycerol) biosynthesis pathway in the control group and the *Cryptosporidium*-positive group (*q* = 0.021; Table 1; Additional file 14).

### The haem biosynthesis pathway is associated with susceptibility to *C. parvum* infection

The MetaCyc pathway relative abundance of PWY-5920: superpathway of haem biosynthesis from glycine, was significantly lower in the control group compared to the *Cryptosporidium*-positive group (*q* = 0.057; Fig. 3B; Additional file 1: Fig. S9). This pathway was attributed to *Escherichia coli* (Table 1; Additional file 14). Otherwise, the rest of the abundances were unclassified in the stratified data.

### Purine nucleotide salvage is associated with susceptibility to *C. parvum* infection and inosine 5-monophosphate degradation is associated with health

The MetaCyc pathway relative abundance of PWY-5695: urate biosynthesis/inosine 5-monophosphate (IMP) degradation was significantly higher in abundance in the control group compared to the *Cryptosporidium*-positive group (*q* = 0.11; Fig. 3E; Additional file 1: Fig. S10). The PWY66-409: superpathway of purine nucleotide salvage pathway abundance was significantly lower in the control group versus the *Cryptosporidium*-positive group (*q* = 0.15; Fig. 3I; Additional file 1: Fig. S10). Multiple species, including *Clostridium perfringens, Enterobacter cloacae complex, Enterococcus faecalis, Escherichia coli, Klebsiella pneumonia* and *Proteus mirabilis* were responsible for the relative abundance of the purine nucleotide salvage pathway in the control group versus the *Cryptosporidium*-positive group (Table 1; Additional file 14). This was also the case for the IMP degradation pathway abundances in the control group, with numerous species of the microbiome contributing to this pathway (Table 1; Additional file 14).

### Other variables impact the taxonomic and functional composition of the microbiome

The factor that had the most profound effect on microbiome species composition was day of sampling. Calves were sampled in the first week of life, however, the day of swabbing within this time frame varied between 1–7 days for the sampled population. Calves that had swabs taken closer to their day of birth (Day 1–3) had significantly different gut microbiomes compared to calves that had swabs taken in the latter part of the week (Day 4–7; Fig. 1D–F, Additional file 1: Fig. S4).

Though the impact of routine antibiotic/anti-cryptosporidial use on the calf microbiome was not the focus of this study, the inclusion of calves that had been treated prior to sampling was unavoidable. This was taken into consideration during selection of controls which were matched as closely as possible to *Cryptosporidium*-positive calves by prophylactic treatment which included Diatrim, Synulox and Halocur, though within farm treatment was not always consistent. To mitigate their effect, these confounding variables were included as fixed effects in the MaAsLin2 analysis so that any significant differences between the control and *Cryptosporidium*-positive groups were a consequence of prospective infection rather than antimicrobial treatment. Regardless of taking treatments into account, the data showed that the composition and metabolic potential of the microbiome were significantly affected by these routine prophylaxes (Additional files 10 and 13).

No significant differences were observed between controls and *Cryptosporidium*-positive calves for the other destratified functional categories that were profiled in HUMAnN3, including biological processes, cellular components, and molecular functions (Additional files 15, 16, 17).

## Discussion

Understanding the features of the calf faecal microbiome that contribute to *Cryptosporidium* susceptibility could inform the development of new therapies and preventative strategies against infection in cattle. Here we conducted a retrospective case–control study in which shotgun metagenomic sequencing was used to determine the taxonomy and functional potential of the faecal microbiome in control and *Cryptosporidium*-positive neonatal calves, prior to infection.

The multivariate analysis of the taxonomy of the faecal microbiome showed that *Veillonella* species were positively associated with the *Cryptosporidium*-positive group (Additional file 1: Fig. S6), however, this may not be a robust association due to the presence of this taxon in a very small number of samples. On the other hand, it has been reported that a higher abundance of *Veillonella* in the faecal microbiome is associated with diarrhoea in calves [31]. The main finding observed in the microbiome composition data showed that there were no robust significant differences in faecal microbiome diversity or relative abundance between the control and *Cryptosporidium*-positive groups. A recent study that examined the 16S rRNA gene sequences of faecal samples of calves that developed non-specific diarrhoea also found no significant differences in microbiome diversity or relative abundance between 12-day-old healthy and pre-scour calves which may further corroborate our findings [35]. Despite no strong significant differences in diversity or taxa relative abundance between the control and *Cryptosporidium*-positive groups, the general composition of the calf faecal microbiota followed the patterns seen in other studies investigating the early calf microbiome. For example, the predominant phyla observed in the first week of life were *Firmicutes*, *Bacteroidetes, Actinobacteria* and *Proteobacteria* [73, 74]. It should be noted that whilst the faecal microbiome is only a marker of the actual microbiota that interacts with *C. parvum* in the small intestine, studies that have directly sequenced the microbiome of the small intestine show similar trends [25, 26].

Though the faecal microbiota did not directly predict the susceptibility of calves towards cryptosporidiosis, the multivariate analysis revealed that specific pathways were associated with the *Cryptosporidium*-positive group. These pathways were related to isoprenoid precursor biosynthesis, haem biosynthesis and purine salvage. The majority of these pathways were attributed to *Escherichia coli*, *Klebsiella pneumoniae* and *Proteus mirabilis*; all species belonging to the *Enterobacteriaceae* family. Though this family was not significantly more abundant in the *Cryptosporidium*-positive group, *Enterobacteriaceae* has been shown to be associated with diarrhoeal disease in calves [31, 34, 37, 38]. It is likely that this trend was not observed in our data as the samples were collected before the onset of infection.

Though the effect size (coefficient value) of the significant pathways was small, it is striking that all of the pathways are also absent in *Cryptosporidium* parasites due to the lack of an apicoplast and traditional mitochondria. With this in mind, components of these microbial pathways could potentially be exploited as targets in the development of novel therapies or preventatives against bovine cryptosporidiosis.

This study showed that the control group had a lower relative abundance of isoprenoid precursor biosynthesis-related pathways in comparison to the *Cryptosporidium*-positive group, suggesting a higher abundance of microbial isoprenoid precursors may lead to increased susceptibility to *C. parvum* infection. There are two isoprenoid precursors; isopentenyl diphosphate (IPP) and its isomer, dimethylallyl diphosphate (DMAPP), that make up a wide variety of biological molecules that are essential for cellular growth in all living organisms. The majority of the significant pathways relate to the MEP pathway which is one of two pathways responsible for the production of isoprenoid precursors. The MEP pathway is the method by which most bacteria, eukaryotic parasites and plants produce isoprenoid precursors [75]. These compounds are used in the biosynthesis of 2-methyl-1,3-butadiene, also known as isoprene. Isoprene is found in myriad isoprenoid compounds including sterols like cholesterol, vitamins A and D, carotenoids, and haem A [76, 77]. In addition, the teichoic acid (glycerol) biosynthesis pathway includes an interaction with isoprenoids in order to synthesise teichoic acid, a structural component of Gram-positive bacteria cell walls [78].

The MEP pathway takes place in the apicoplast of apicomplexans such as *Plasmodium* and *Toxoplasma* [79]. However, *Cryptosporidium* lacks an apicoplast and as a result is void of the MEP pathway [80]. Though the MEP pathway is absent in *C. parvum* parasites, it has been shown that the parasite encodes enzymes connected to the use of isoprenoid precursors, indicating that *Cryptosporidium* must scavenge the isoprenoid precursors from an external source [81]. Some have suggested that *Cryptosporidium* exploits the production of isoprenoid precursors from the mammalian host cells which are generated via the MVA pathway [79]. The MVA pathway is the method by which most eukaryotes, Archaea, and some bacteria produce isoprenoid precursors [75]. Indeed, a component of the bacterial MVA pathway was also significantly lower in the control group versus the *Cryptosporidium*-positive group in our study. This suggests that *C. parvum* may exploit both MEP and MVA pathways of the bacterial microbiota. But whether *Cryptosporidium* scavenges isoprenoid precursors from the host, the microbiome or both is unknown. In fact, the inhibition of the MVA pathway of host cells in vitro has been shown to reduce growth of *C. parvum* infection in HCT-8 cells using the statin, Itavastatin [82]. This outcome in conjunction with the results of our study would suggest that *C. parvum* may use a combination of host MVA and microbial MEP and MVA pathways in order to scavenge sufficient supplies of IPP. If this were the case, this may imply that the difficulties of culturing *Cryptosporidium* in vitro are due to a lack of bacterial isoprenoid precursors to scavenge and thus introducing these isoprenoid precursors could improve in vitro infection rates for experimental research.

The superpathway of haem biosynthesis was significantly lower in abundance in the control group compared to the *Cryptosporidium*-positive group. This suggests that calves with a higher relative abundance of microbial haem pathways are more susceptible to *C. parvum* infection. Like the apicoplast, a traditional mitochondrion is lacking in *Cryptosporidium*, along with the ability to synthesise haem. Unlike other apicomplexan parasites which exhibit multiple cytochromes, *Cryptosporidium* expresses one haem-containing enzyme of unknown function, suggesting that *Cryptosporidium* has some requirement for haem though it may be minimal [83]. A possible function of this singular enzyme could be sterol manufacture as this is the only process that is utterly haem-dependent and found in most eukaryotes [84]. As previously mentioned, sterol production requires isoprenoid precursors, meaning that haem interacts indirectly with the MEP/MVA pathways. Indeed, haem B, may be converted to other haem derivatives such as haem A and O by transfer of farnesyl groups, a product of the MEP pathway, illustrating another pathway that interacts with isoprenoid metabolites [76]. Though the haem requirements of *Cryptosporidium* may be minimal, this singular enzyme could be inhibited to reduce *Cryptosporidium* infection in calves.

The purine salvage pathway (used for the production of purine nucleotides from recycled purine bases) was found to be significantly lower in abundance in the control group, whereas the IMP degradation pathway was significantly higher in the control group compared to the *Cryptosporidium*-positive group. Purine nucleotides are essential for the survival of *C. parvum* as like any living organism, they require an energy source and the constituents to assemble DNA and RNA. However, *C. parvum* is not able to synthesise purines de novo but possesses purine salvage mechanisms [85]. It has been proposed that when this pathway is ablated in *C. parvum*, the parasite is able to take advantage of host cell purine salvage pathways and obtain purine nucleotides from the cytoplasm of the host cell in which it resides [86]. Though *C. parvum* may have the capacity to manipulate the host to exploit its purine salvage pathways, our data suggests that calves that have a higher relative abundance of purine salvage pathways within the microbiota are at higher risk of becoming infected with *C. parvum*. This implies that *Cryptosporidium* may be able to exploit the purine salvage pathways of the microbiome in conjunction with host salvage mechanisms.

The bacterial purine salvage pathway may be a potential target to inhibit *C. parvum* infection. A study investigating the effect of purine nucleosides on in vitro* C. parvum* infection showed that inosine improved the growth of the parasite in THP-1 cells, particularly the trophic stages. This shows the importance of purine metabolism in *C. parvum* [87]. It has been suggested that inhibition of activities in the pathway between adenosine and guanosine monophosphate (GMP) (important molecules in energy metabolism) in *Cryptosporidium* would lead to killing of the parasite as it relies upon this single pathway to produce GMP [88]. Comparable to haem biosynthesis, it appears that *Cryptosporidium* has simplified its mechanism for purine procurement. The enzyme, IMP dehydrogenase (IMPDH), catalyses the rate-limiting step that converts exogenous purines such as adenosine into GMP [89]. Therefore, it has been proposed that IMPDH could be a potential candidate for drug development against *C. parvum* infection. Indeed, one study has already demonstrated the antiparasitic properties of IMPDH inhibitors in a mouse model of cryptosporidiosis [90].

If *C. parvum* does scavenge metabolites from the microbiome, we would like to suggest the probable mechanisms by which the parasite may interact with the host and microbiota to procure these compounds. Firstly, we hypothesise that the parasite may uptake microbiota-derived metabolites prior to invading the enterocytes, to meet energy requirements for parasite motility and cell invasion. Alternatively, we suggest that the epithelial cells uptake the bacterial metabolites and the parasite retrieves these second-hand compounds, following cell invasion. Some studies have already investigated the impact of microbiota-derived metabolites on *Cryptosporidium* infection in mice [91, 92]. One study demonstrated that medium- or long-chain saturated fatty acids inhibited the growth of *C. parvum*, whilst long-chain polyunsaturated fatty acids promoted *C. parvum* infection in mice [92]. Another recent study shows that indole has an inhibitory effect on the growth of *C. parvum* in mice and on host mitochondrial respiration in HCT-8 cells which could affect the parasites ability to scavenge essential metabolites from the host [91]. These studies further endorse the described approach as a potential therapeutic avenue against cryptosporidiosis, however further investigation is required to ascertain the interaction between *Cryptosporidium* and the microbiota-derived metabolites in cattle.

The findings of this study lead us to suggest potential therapeutic or preventative strategies against *Cryptosporidium* infection such as compounds that directly inhibit these microbial pathways or pre/pro/post-biotics/FMT therapies that minimise or replace the microbes contributing to them. If effective, the main dilemma of inhibiting microbial pathways or manipulating the microbiome in any way is the potential negative impact on the microbiome, and in turn the host. Further research is required to explore these recommendations.

### Limitations

The study design inherently has limitations that we will discuss here.

The use of LFT to confirm *C. parvum* infection in the calves that developed diarrhoea may not have the highest sensitivity/specificity of the detection methods that are available. However, in terms of cost, turnaround time and user friendliness, it is a practical approach for testing large numbers of samples for multiple causes of infectious diarrhoea in a farm environment. The LFT in combination with the veterinary clinical diagnosis of diarrhoea is considered sufficient clinical diagnostic criteria for cryptosporidiosis. With additional resources a qPCR approach could have been employed to improve the confidence in the diagnosis, however, this technique is much more costly, time-consuming and labour-intensive.

In addition, the potential for the presence of other pathogens that were not tested for is a possibility. This could be described as an unknown confounding variable. However, we tested for the three other major infectious causes of calf diarrhoea using the LFT kit in the calves that developed diarrhoea, in order to select calves that tested positive for *C. parvum* or *C. parvum* co-infection. Whilst two of the case calves tested positive for both *C. parvum* and *Rotavirus*, it is not uncommon for *C. parvum* to be detected alongside other pathogens due to the associated dysbiosis increasing calf susceptibility to infection.

The provision of antibiotics and other treatments at birth to several enrolled calves was unavoidable as the study was conducted on commercial dairies. The compositional and functional differences of the microbiome between control and *Cryptosporidium*-positive calves may have been more pronounced without these routine treatments as their ability to alter the microbiome may have had a masking effect. Though it is highly likely that the results of the study may have differed had the calves not received treatment, this aspect of the study is also a strength as it improves the external validity of the study findings.

Finally, we were only able to show associations between susceptibility to infection and features of the microbiome since the study was observational. Consequently, any conclusions drawn from this study will require further investigation. In vitro research in a *C. parvum*-bacteria-host cell co-culture system with pathway inhibitors could be an initial approach to ascertaining the importance of bacterial pathways in *C. parvum* infection.

## Conclusion

In summary, we conclude that *C. parvum* may be able to harness the isoprenoid precursor biosynthesis, haem biosynthesis and purine salvage pathways of the host microbiota in order to survive and calves that are more abundant in these microbiota-associated pathways may be more susceptible to *Cryptosporidium* infection. This could be important for development of novel treatments or preventative strategies against bovine cryptosporidiosis as components of these pathways could be exploited as potential therapeutic targets.

### Supplementary Information

**Additional file 1. Figure S1.** Experimental study design. One faecal swab sample was collected from each calf enrolled during week 1 of life from three farms (n=346). Calves were observed for signs of diarrhoeal disease amongst other health monitoring checks. Calves that exhibited diarrhoea had a swab taken at the point of scour and a LFT to determine the cause of diarrhoea. Healthy control calves (n = 33) and calves that tested positive for *C. parvum* (n = 32) were selected matching for age, sex, breed, and prior treatment where possible. Week 1 faecal swab samples from the selected control and *Cryptosporidium*-positive calves were extracted and DNA that did not meet CGR QC requirements was excluded from the study (n = 5). The final 60 DNA samples underwent shotgun metagenomic sequencing, processing, and analysis. **Figure S2.** The total number of reads obtained for each sample. **Figure S3.** The distribution of trimmed read lengths for the forward (R1), reverse (R2) and singlet (R0) reads. **Figure S4.** Alpha and beta diversity of disease status and sample collection day group interaction. **A** Species richness in control Day 1–3 (n = 7) and *Cryptosporidium*-positive Day 1–3 (n = 13) groups; T-test, *p* = 0.21, and control Day 4–7 (n = 23) and *Cryptosporidium*-positive Day 4–7 (n = 17) groups; T-test, *p* = 0.85. **B** Shannon index of control Day 1–3 (n = 7) and *Cryptosporidium*-positive Day 1–3 (n = 13) groups; Wilcoxon, *p* = 0.081, and control Day 4–7 (n = 23) and *Cryptosporidium*-positive Day 4–7 (n = 17) groups; Wilcoxon, *p* = 0.67. **C** Bray Curtis PCoA ordination of control Day 1–3 (n = 7) and *Cryptosporidium*-positive Day 1–3 (n = 13) groups; PERMANOVA, *p* = 0.45, and control Day 4–7 (n = 23) and *Cryptosporidium*-positive Day 4–7 (n = 17) groups; PERMANOVA, *p* = 0.82. **Figure S5.** Per sample relative abundance. **A** Phyla relative abundance (≥1%) per sample in chronological order of day of sampling. **B** Genera relative abundance (≥1%) per sample in chronological order of day of sampling. **Figure S6.** Significant Taxa. **A** Significant TSS normalised Bracken species relative abundance of the control and *Cryptosporidium*-positive groups shows that *Veillonella rodentium* relative abundance is significantly lower in the control group (n = 1) versus the *Cryptosporidium*-positive group (n = 8); *q* = 0.21. **B** Significant TSS normalised MetaPhlAn2 species relative abundance of the control and *Cryptosporidium*-positive groups shows that *Veillonella* sp. CAG 933 relative abundance is significantly lower in the control group (n = 13) versus the *Cryptosporidium*-positive group (n = 10); *q* = 0.16. **Figure S7.** MetaCyc Pathway ID and Q-values of significant pathway relative abundances between the control and the *Cryptosporidium*-positive groups. **Figure S8.** Flowchart of the MEP pathway and downstream processes. Based on the MetaCyc database pathway [93]. **Figure S9.** Flowchart of the superpathway of haem biosynthesis from glycine. Based on the MetaCyc database pathway [93]. **Figure S10.** Flowchart of the purine nucleotide salvage pathway and inosine-5-monophosphate degradation pathway. Based on the MetaCyc database pathway [93]. **Table S1.** Percentage of reads retained after removal of host reads.

**Additional file 2.** Taxonomic Functional Profiling Custom Script.

**Additional file 3.** Taxonomic Rank Parse Script.

**Additional file 4.** Bracken Species Relative Abundance Table.

**Additional file 5.** MetaPhlAn2 Species Relative Abundance Table.

**Additional file 6.** Metadata File.

**Additional file 7.** R Analysis Code.

**Additional file 8.** Bracken Phyla MaAsLin2 Results Table.

**Additional file 9.** Bracken Genus MaAsLin2 Results Table.

**Additional file 10.** Bracken Species Maaslin2 Results Table.

**Additional file 11.** MetaPhlAn2 Species Maaslin2 Results Table.

**Additional file 12.** Destratified Pathway Abundance Table.

**Additional file 13.** Pathway Abundance MaAsLin2 Results Table.

**Additional file 14.** Stratified Pathway Abundance Species Assignment Table.

**Additional file 15.** Biological Processes MaAsLin2 Results Table.

**Additional file 16.** Cellular Components MaAsLin2 Results Table.

**Additional file 17.** Molecular Functions MaAsLin2 Results Table.

## Data Availability

The raw sequence data can be found in the NCBI repository in BioProject: PRJNA935534.

## Abbreviations

GIT: Gastrointestinal tract
FMT: Faecal microbiota transplantation
LFT: Lateral flow test
BCS: Body condition score
CGR: Centre of Genomic Research
CPM: Counts per million reads
MEP: Methylerythritol phosphate
MVA: Mevalonate
IMP: Inosine 5-monophosphate
IPP: Isopentenyl diphosphate
DMAPP: Dimethylallyl diphosphate
GMP: Guanosine monophosphate
IMPDH: Inosine 5-monophosphate dehydrogenase
