# Supplementary material for: Specific pathway abundances in the neonatal calf faecal microbiome are associated with susceptibility to Cryptosporidium parvum infection: a metagenomic analysis
Source: Anim Microbiome. 2023 Sep 12;5:43. doi: 10.1186/s42523-023-00265-5 (PMC10496319; doi:10.1186/s42523-023-00265-5)
Supplement: Supplementary file 1 — Additional file 1. Figure S1. Experimental study design. One faecal swab sample was collected from each calf enrolled during week 1 of life from three farms (n=346). Calves were observed for signs of diarrhoeal disease amongst other health monitoring checks. Calves that exhibited diarrhoea had a swab taken at the point of scour and a LFT to determine the cause of diarrhoea. Healthy control calves (n = 33) and calves that tested positive for C. parvum (n = 32) were selected matching for age, sex, breed, and prior treatment where possible. Week 1 faecal swab samples from the selected control and Cryptosporidium-positive calves were extracted and DNA that did not meet CGR QC requirements was excluded from the study (n = 5). The final 60 DNA samples underwent shotgun metagenomic sequencing, processing, and analysis. Figure S2. The total number of reads obtained for each sample. Figure S3. The distribution of trimmed read lengths for the forward (R1), reverse (R2) and singlet (R0) reads. Figure S4. Alpha and beta diversity of disease status and sample collection day group interaction. A Species richness in control Day 1–3 (n = 7) and Cryptosporidium-positive Day 1–3 (n = 13) groups; T-test, p = 0.21, and control Day 4–7 (n = 23) and Cryptosporidium-positive Day 4–7 (n = 17) groups; T-test, p = 0.85. B Shannon index of control Day 1–3 (n = 7) and Cryptosporidium-positive Day 1–3 (n = 13) groups; Wilcoxon, p = 0.081, and control Day 4–7 (n = 23) and Cryptosporidium-positive Day 4–7 (n = 17) groups; Wilcoxon, p = 0.67. C Bray Curtis PCoA ordination of control Day 1–3 (n = 7) and Cryptosporidium-positive Day 1–3 (n = 13) groups; PERMANOVA, p = 0.45, and control Day 4–7 (n = 23) and Cryptosporidium-positive Day 4–7 (n = 17) groups; PERMANOVA, p = 0.82. Figure S5. Per sample relative abundance. A Phyla relative abundance (≥1%) per sample in chronological order of day of sampling. B Genera relative abundance (≥1%) per sample in chronological order of day of sampling. Figure S6. [file 42523_2023_265_MOESM1_ESM.pptx]

## Slide 1
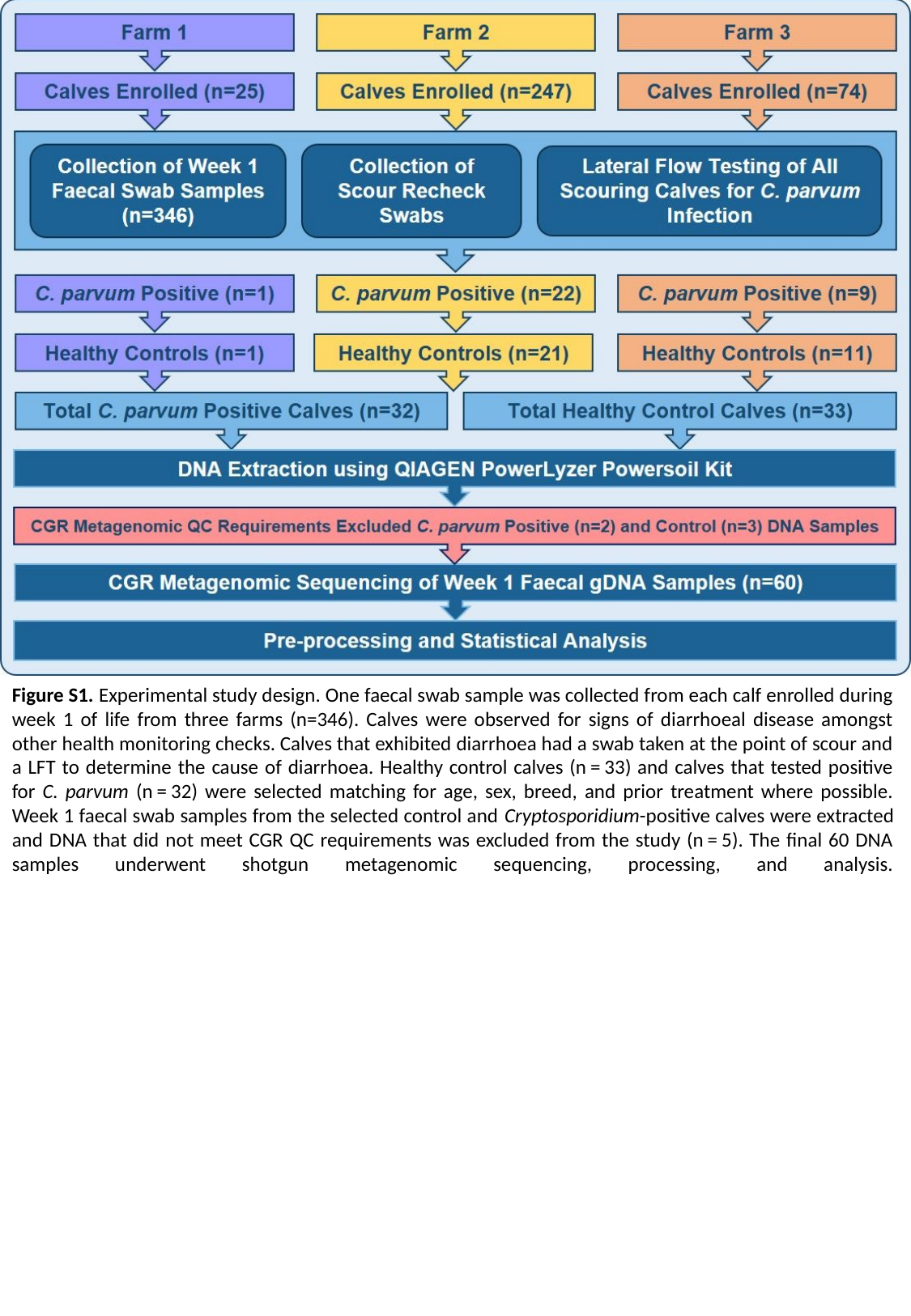

Figure S1. Experimental study design. One faecal swab sample was collected from each calf enrolled during week 1 of life from three farms (n=346). Calves were observed for signs of diarrhoeal disease amongst other health monitoring checks. Calves that exhibited diarrhoea had a swab taken at the point of scour and a LFT to determine the cause of diarrhoea. Healthy control calves (n = 33) and calves that tested positive for C. parvum (n = 32) were selected matching for age, sex, breed, and prior treatment where possible. Week 1 faecal swab samples from the selected control and Cryptosporidium-positive calves were extracted and DNA that did not meet CGR QC requirements was excluded from the study (n = 5). The final 60 DNA samples underwent shotgun metagenomic sequencing, processing, and analysis.

## Slide 2
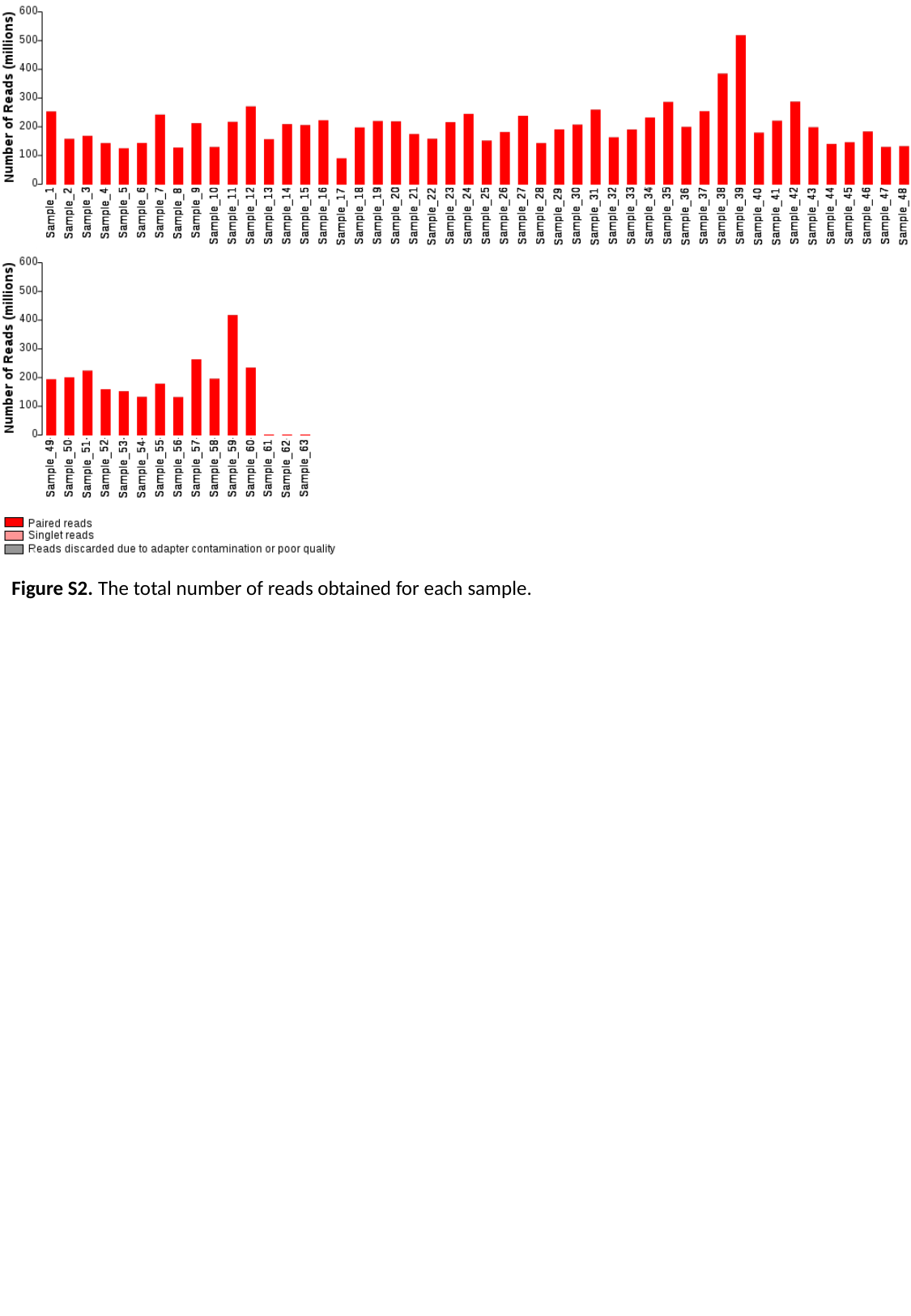

Figure S2. The total number of reads obtained for each sample.

## Slide 3
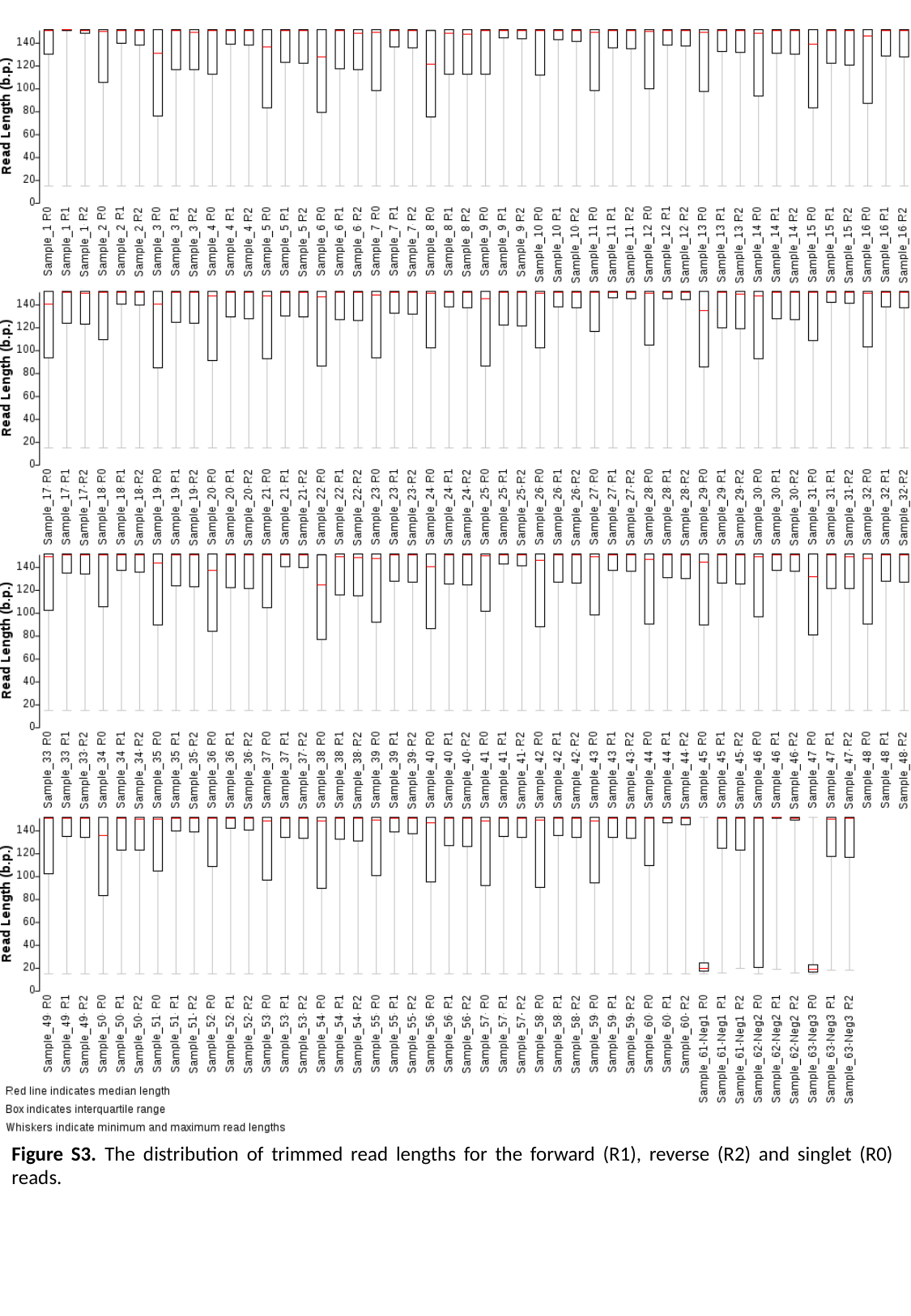

Figure S3. The distribution of trimmed read lengths for the forward (R1), reverse (R2) and singlet (R0) reads.

## Slide 4
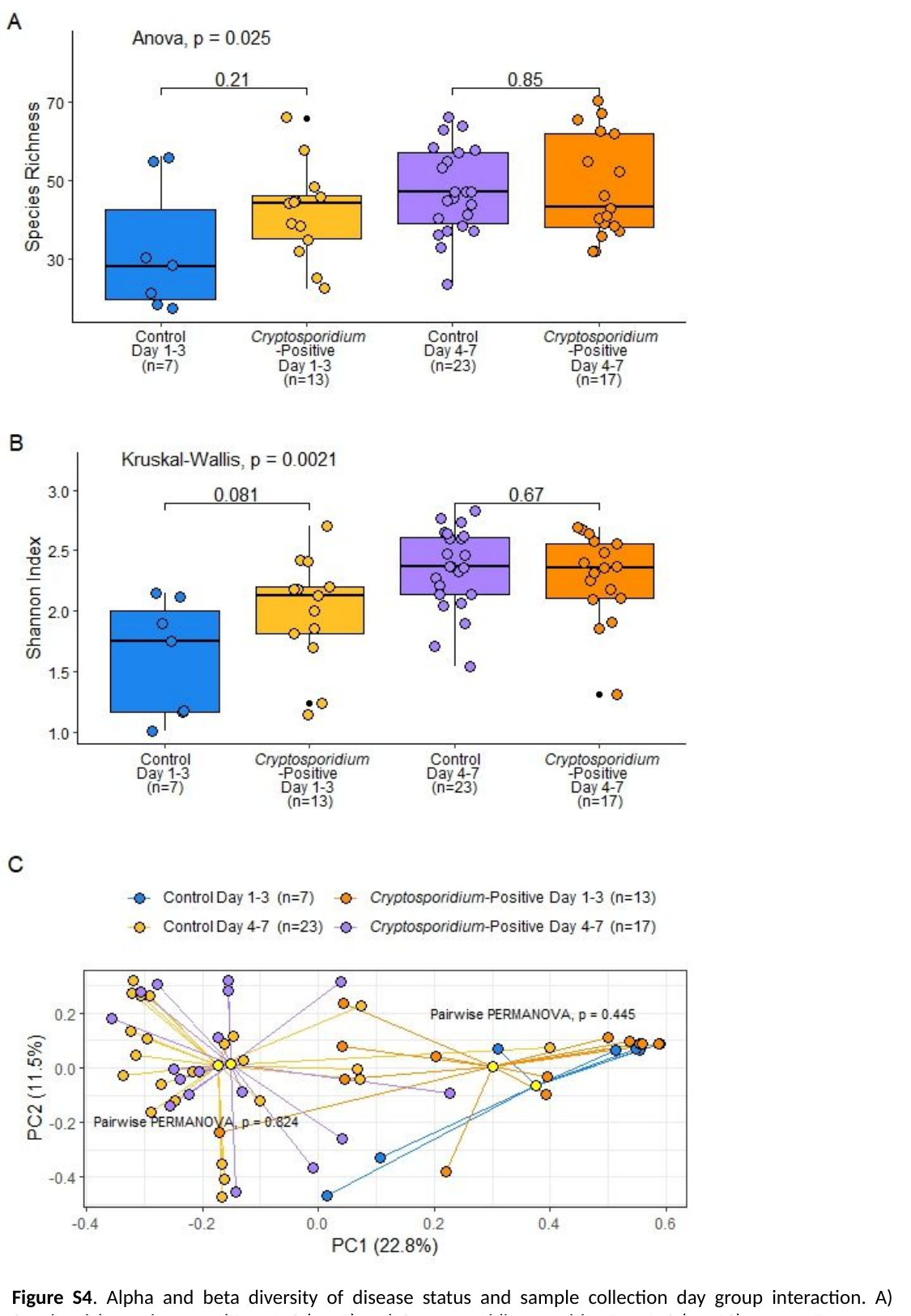

Figure S4. Alpha and beta diversity of disease status and sample collection day group interaction. A) Species richness in control Day 1–3 (n = 7) and Cryptosporidium-positive Day 1–3 (n = 13) groups; T-test, p = 0.21, and control Day 4–7 (n = 23) and Cryptosporidium-positive Day 4–7 (n = 17) groups; T-test, p = 0.85. B) Shannon index of control Day 1–3 (n = 7) and Cryptosporidium-positive Day 1–3 (n = 13) groups; Wilcoxon, p = 0.081, and control Day 4–7 (n = 23) and Cryptosporidium-positive Day 4–7 (n = 17) groups; Wilcoxon, p = 0.67. C) Bray Curtis PCoA ordination of control Day 1–3 (n = 7) and Cryptosporidium-positive Day 1–3 (n = 13) groups; PERMANOVA, p = 0.45, and control Day 4–7 (n = 23) and Cryptosporidium-positive Day 4–7 (n = 17) groups; PERMANOVA, p = 0.82.

## Slide 5
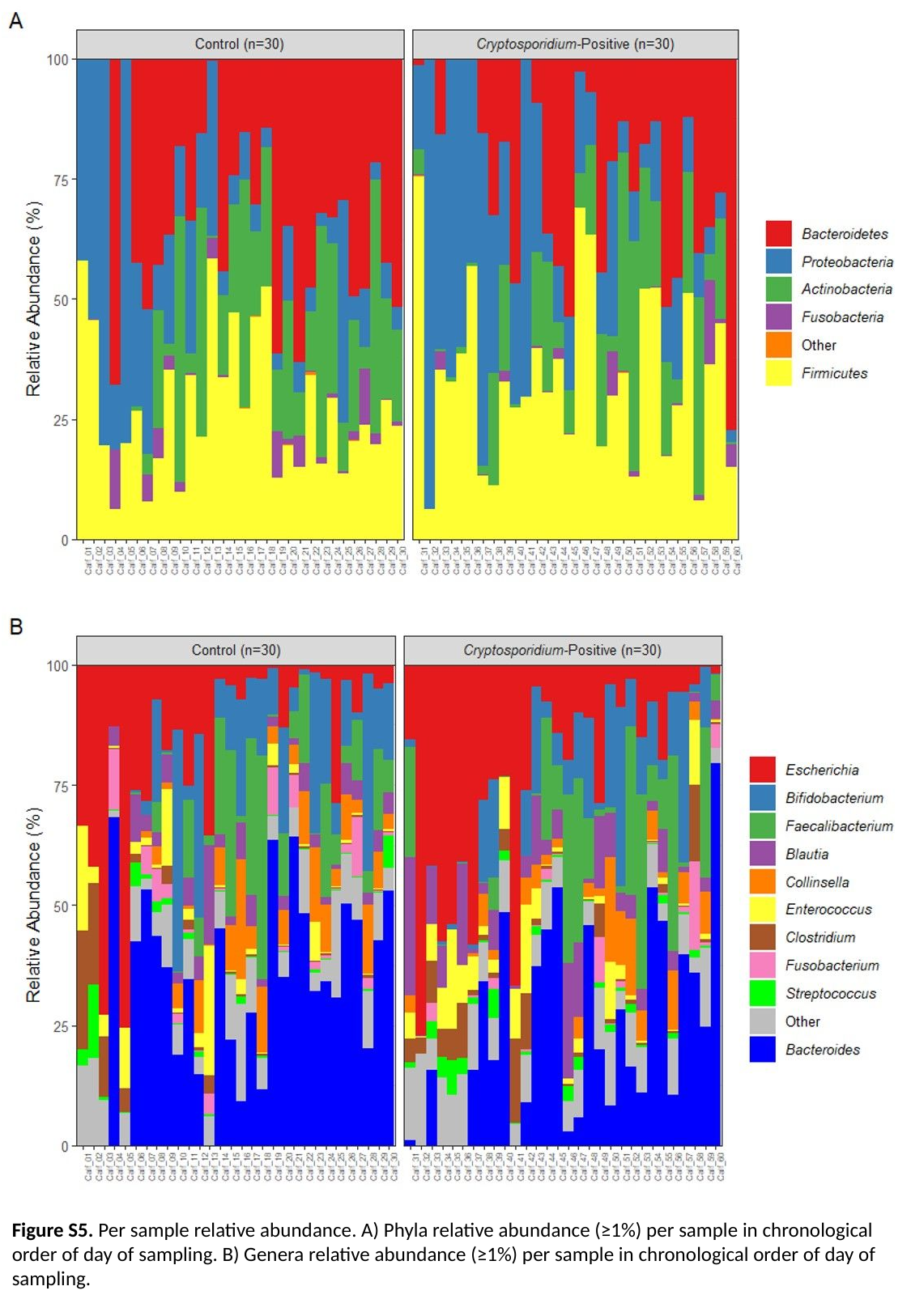

Figure S5. Per sample relative abundance. A) Phyla relative abundance (≥1%) per sample in chronological order of day of sampling. B) Genera relative abundance (≥1%) per sample in chronological order of day of sampling.

## Slide 6
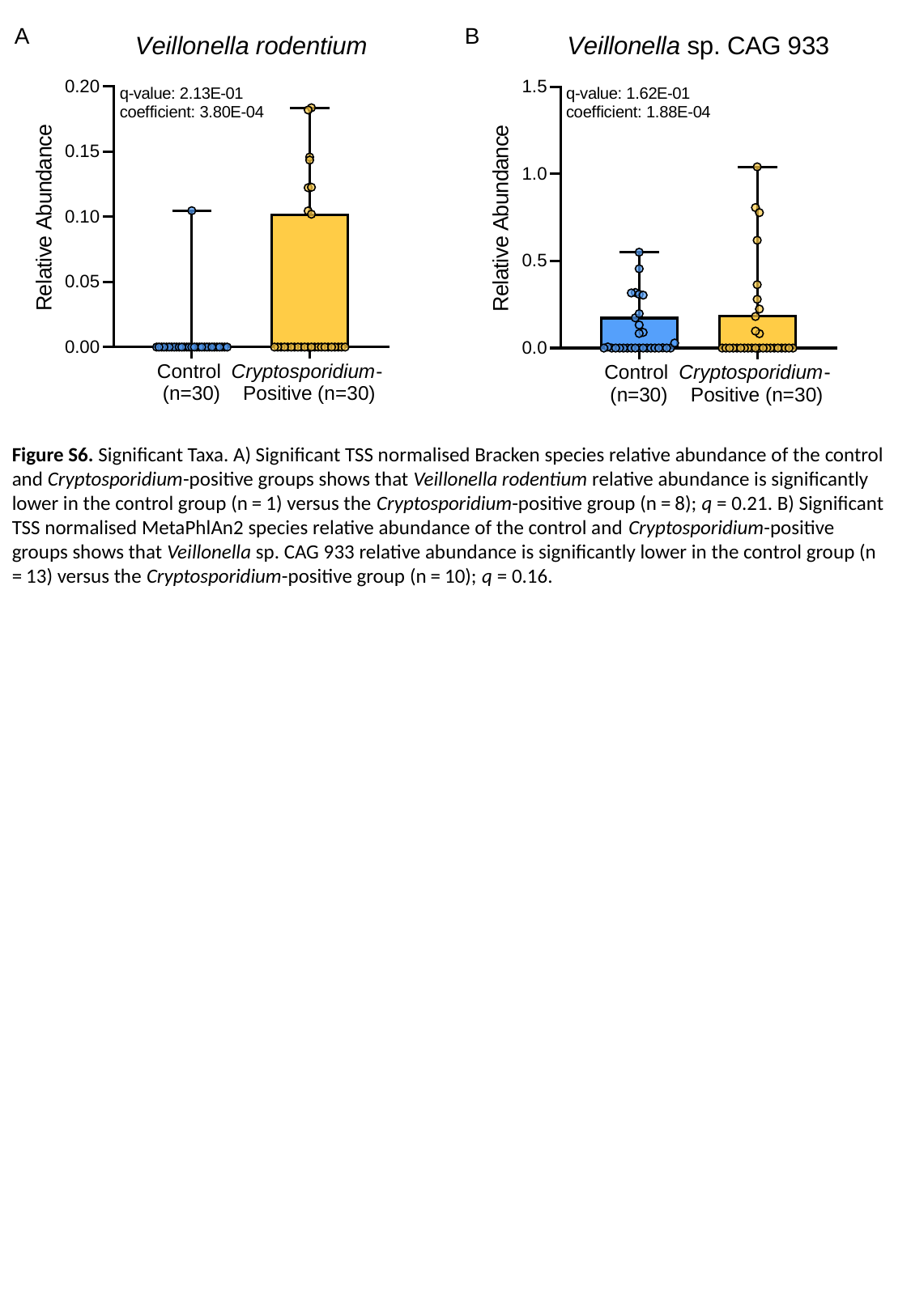

Figure S6. Significant Taxa. A) Significant TSS normalised Bracken species relative abundance of the control and Cryptosporidium-positive groups shows that Veillonella rodentium relative abundance is significantly lower in the control group (n = 1) versus the Cryptosporidium-positive group (n = 8); q = 0.21. B) Significant TSS normalised MetaPhlAn2 species relative abundance of the control and Cryptosporidium-positive groups shows that Veillonella sp. CAG 933 relative abundance is significantly lower in the control group (n = 13) versus the Cryptosporidium-positive group (n = 10); q = 0.16.

## Slide 7
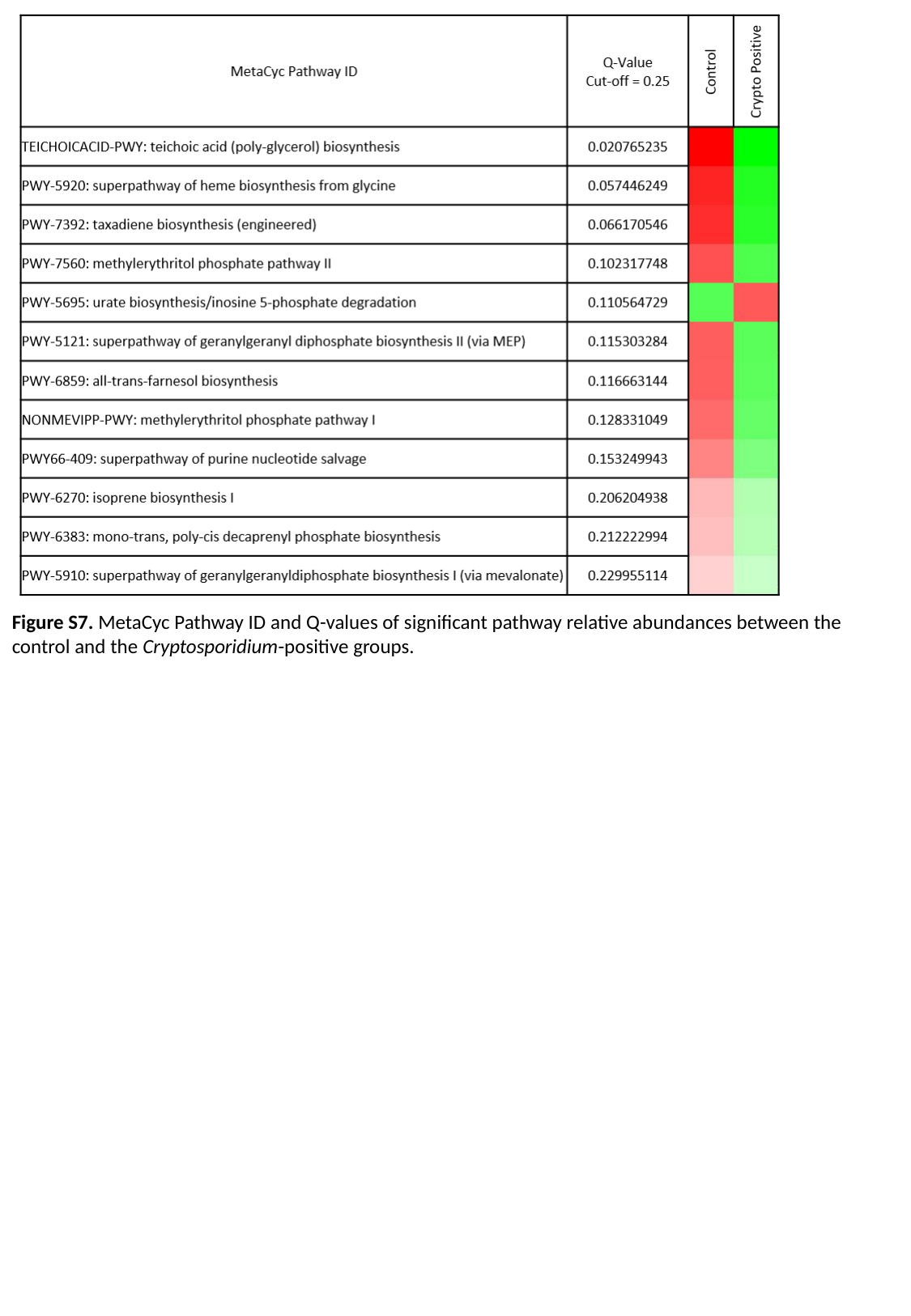

Figure S7. MetaCyc Pathway ID and Q-values of significant pathway relative abundances between the control and the Cryptosporidium-positive groups.

## Slide 8
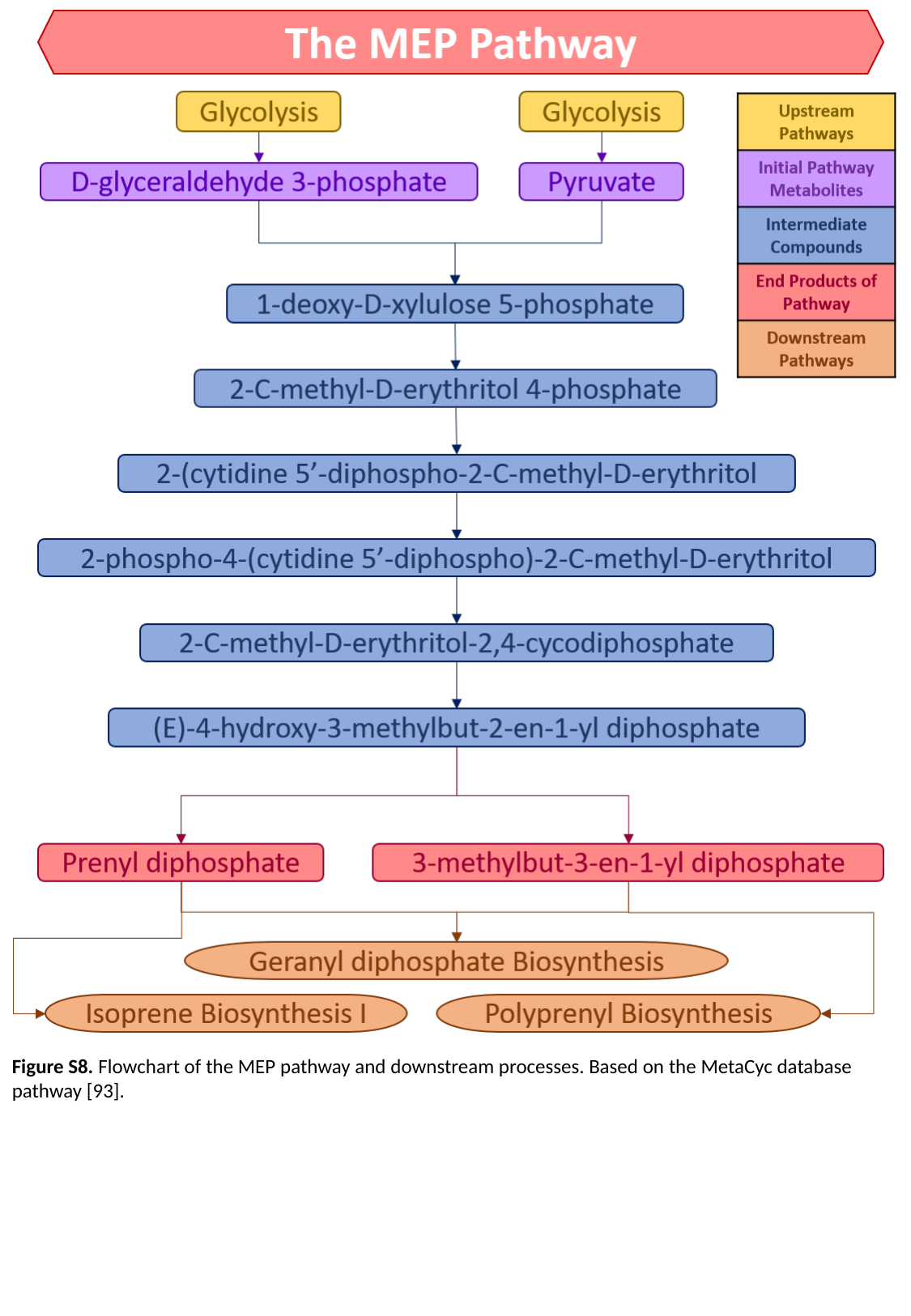

Figure S8. Flowchart of the MEP pathway and downstream processes. Based on the MetaCyc database pathway [93].

## Slide 9
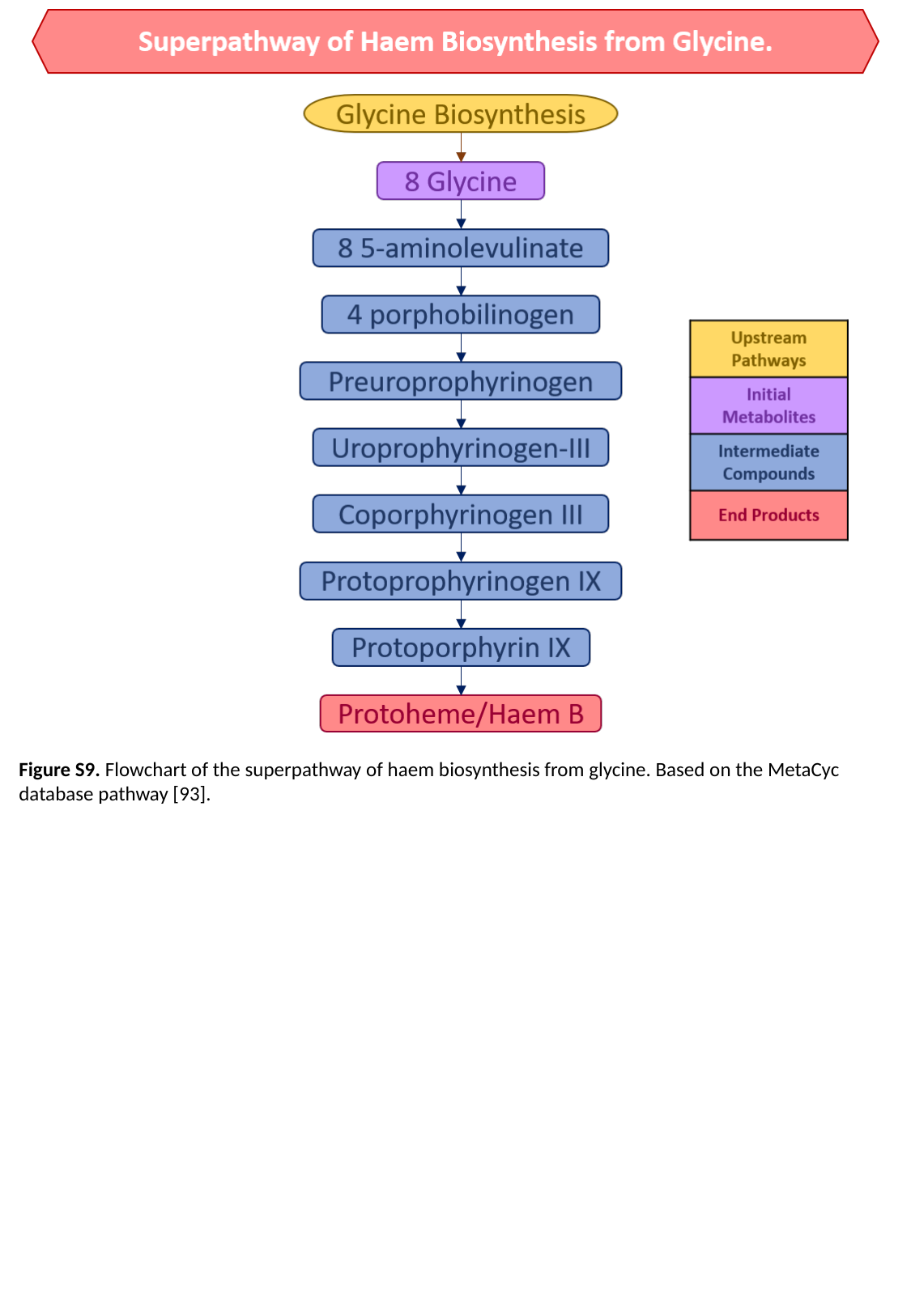

Figure S9. Flowchart of the superpathway of haem biosynthesis from glycine. Based on the MetaCyc database pathway [93].

## Slide 10
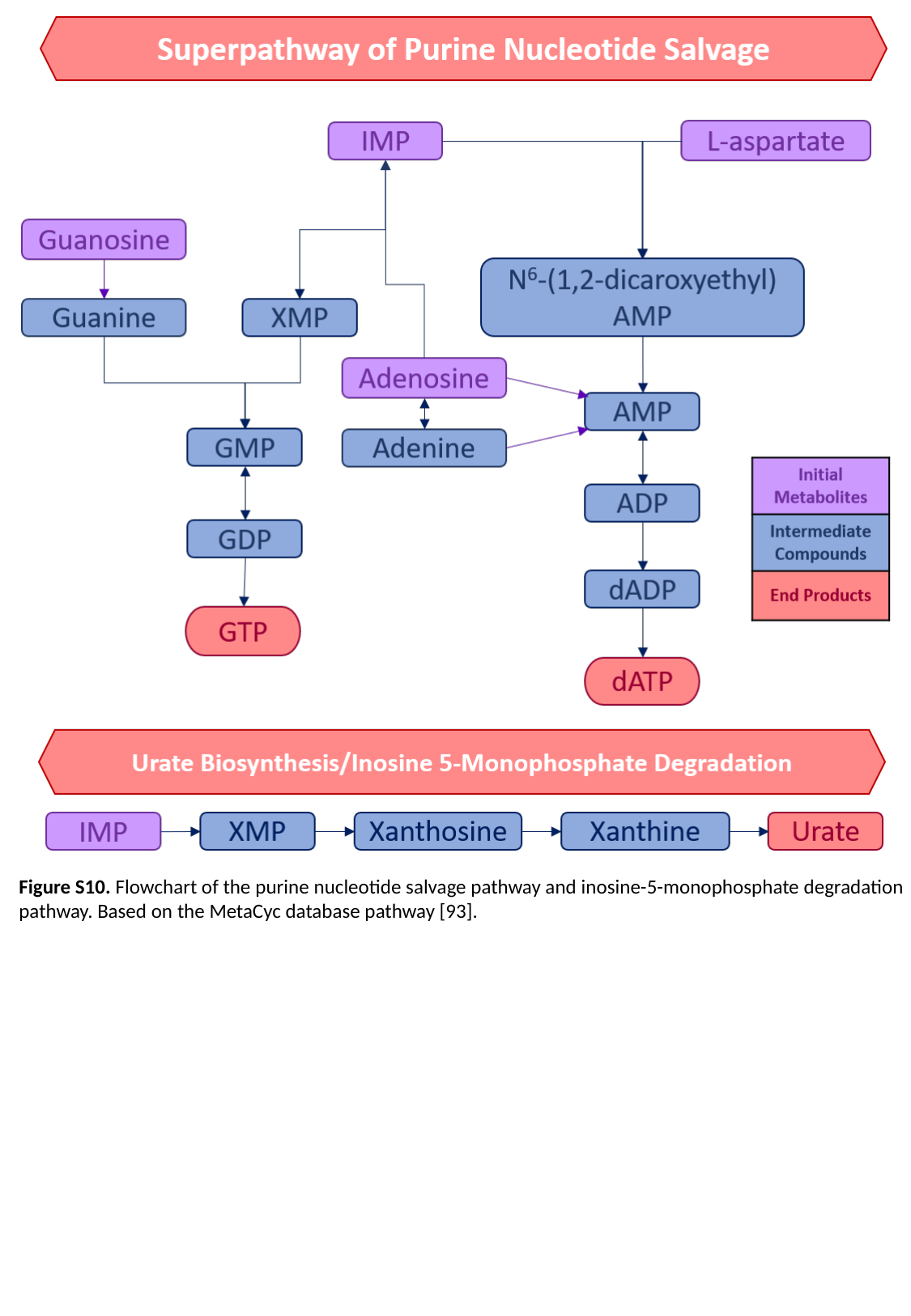

Figure S10. Flowchart of the purine nucleotide salvage pathway and inosine-5-monophosphate degradation pathway. Based on the MetaCyc database pathway [93].

## Slide 11
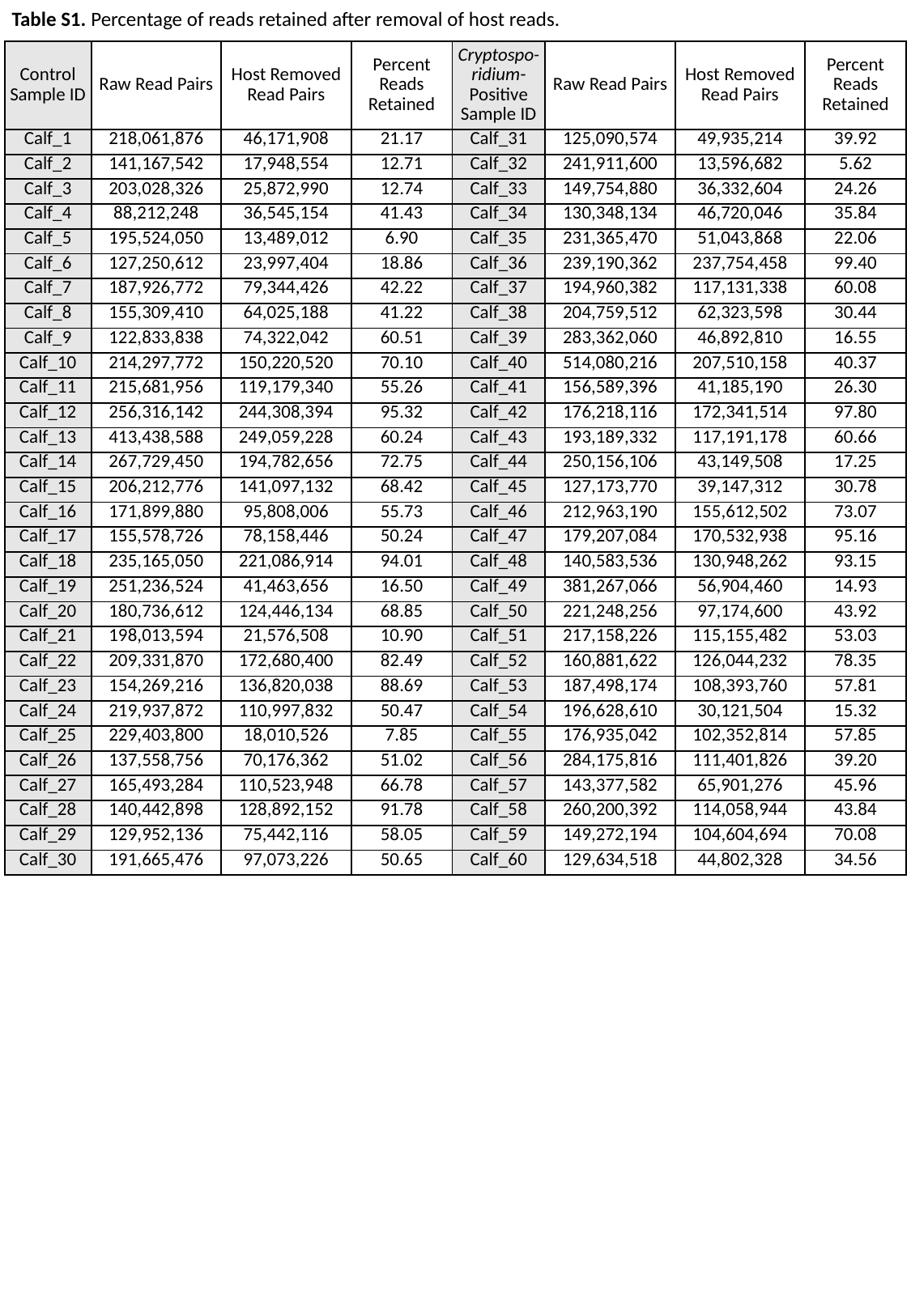

Table S1. Percentage of reads retained after removal of host reads.
| Control Sample ID | Raw Read Pairs | Host Removed Read Pairs | Percent Reads Retained | Cryptospo-ridium-Positive Sample ID | Raw Read Pairs | Host Removed Read Pairs | Percent Reads Retained |
| --- | --- | --- | --- | --- | --- | --- | --- |
| Calf\_1 | 218,061,876 | 46,171,908 | 21.17 | Calf\_31 | 125,090,574 | 49,935,214 | 39.92 |
| Calf\_2 | 141,167,542 | 17,948,554 | 12.71 | Calf\_32 | 241,911,600 | 13,596,682 | 5.62 |
| Calf\_3 | 203,028,326 | 25,872,990 | 12.74 | Calf\_33 | 149,754,880 | 36,332,604 | 24.26 |
| Calf\_4 | 88,212,248 | 36,545,154 | 41.43 | Calf\_34 | 130,348,134 | 46,720,046 | 35.84 |
| Calf\_5 | 195,524,050 | 13,489,012 | 6.90 | Calf\_35 | 231,365,470 | 51,043,868 | 22.06 |
| Calf\_6 | 127,250,612 | 23,997,404 | 18.86 | Calf\_36 | 239,190,362 | 237,754,458 | 99.40 |
| Calf\_7 | 187,926,772 | 79,344,426 | 42.22 | Calf\_37 | 194,960,382 | 117,131,338 | 60.08 |
| Calf\_8 | 155,309,410 | 64,025,188 | 41.22 | Calf\_38 | 204,759,512 | 62,323,598 | 30.44 |
| Calf\_9 | 122,833,838 | 74,322,042 | 60.51 | Calf\_39 | 283,362,060 | 46,892,810 | 16.55 |
| Calf\_10 | 214,297,772 | 150,220,520 | 70.10 | Calf\_40 | 514,080,216 | 207,510,158 | 40.37 |
| Calf\_11 | 215,681,956 | 119,179,340 | 55.26 | Calf\_41 | 156,589,396 | 41,185,190 | 26.30 |
| Calf\_12 | 256,316,142 | 244,308,394 | 95.32 | Calf\_42 | 176,218,116 | 172,341,514 | 97.80 |
| Calf\_13 | 413,438,588 | 249,059,228 | 60.24 | Calf\_43 | 193,189,332 | 117,191,178 | 60.66 |
| Calf\_14 | 267,729,450 | 194,782,656 | 72.75 | Calf\_44 | 250,156,106 | 43,149,508 | 17.25 |
| Calf\_15 | 206,212,776 | 141,097,132 | 68.42 | Calf\_45 | 127,173,770 | 39,147,312 | 30.78 |
| Calf\_16 | 171,899,880 | 95,808,006 | 55.73 | Calf\_46 | 212,963,190 | 155,612,502 | 73.07 |
| Calf\_17 | 155,578,726 | 78,158,446 | 50.24 | Calf\_47 | 179,207,084 | 170,532,938 | 95.16 |
| Calf\_18 | 235,165,050 | 221,086,914 | 94.01 | Calf\_48 | 140,583,536 | 130,948,262 | 93.15 |
| Calf\_19 | 251,236,524 | 41,463,656 | 16.50 | Calf\_49 | 381,267,066 | 56,904,460 | 14.93 |
| Calf\_20 | 180,736,612 | 124,446,134 | 68.85 | Calf\_50 | 221,248,256 | 97,174,600 | 43.92 |
| Calf\_21 | 198,013,594 | 21,576,508 | 10.90 | Calf\_51 | 217,158,226 | 115,155,482 | 53.03 |
| Calf\_22 | 209,331,870 | 172,680,400 | 82.49 | Calf\_52 | 160,881,622 | 126,044,232 | 78.35 |
| Calf\_23 | 154,269,216 | 136,820,038 | 88.69 | Calf\_53 | 187,498,174 | 108,393,760 | 57.81 |
| Calf\_24 | 219,937,872 | 110,997,832 | 50.47 | Calf\_54 | 196,628,610 | 30,121,504 | 15.32 |
| Calf\_25 | 229,403,800 | 18,010,526 | 7.85 | Calf\_55 | 176,935,042 | 102,352,814 | 57.85 |
| Calf\_26 | 137,558,756 | 70,176,362 | 51.02 | Calf\_56 | 284,175,816 | 111,401,826 | 39.20 |
| Calf\_27 | 165,493,284 | 110,523,948 | 66.78 | Calf\_57 | 143,377,582 | 65,901,276 | 45.96 |
| Calf\_28 | 140,442,898 | 128,892,152 | 91.78 | Calf\_58 | 260,200,392 | 114,058,944 | 43.84 |
| Calf\_29 | 129,952,136 | 75,442,116 | 58.05 | Calf\_59 | 149,272,194 | 104,604,694 | 70.08 |
| Calf\_30 | 191,665,476 | 97,073,226 | 50.65 | Calf\_60 | 129,634,518 | 44,802,328 | 34.56 |
