# Supplementary material for: Specific pathway abundances in the neonatal calf faecal microbiome are associated with susceptibility to Cryptosporidium parvum infection: a metagenomic analysis
Source: Anim Microbiome. 2023 Sep 12;5:43. doi: 10.1186/s42523-023-00265-5 (PMC10496319; doi:10.1186/s42523-023-00265-5)
Supplement: Supplementary file 7 — Additional file 7. R Analysis Code. [file 42523_2023_265_MOESM7_ESM.pdf]

Specific pathway abundances in the neonatal calf faecal microbiome are associated with susceptibility to *Cryptosporidium parvum* infection: a metagenomic analysis

Hares et al.

This document contains all statistical analysis and figures produced in RStudio for this study.

Alpha and Beta Diversity Analysis

```
library(vegan)
library(ggplot2)
library(ape)
library(ggpubr)
library(ggsignif)
library(ggtext)
library(glue)
library(scales)
library(markdown)

bracken <- read.delim (file = 'S.percent.txt', row.names = 1)
metadata <- read.delim (file = 'metadata.txt', row.names = 1)
richness <- specnumber(bracken)
shannon <- diversity(bracken)
data_alphadiv <- cbind(metadata, richness, shannon)

#Figure 1A
CP_Richness <- ggplot(data_alphadiv, aes(x=Crypto, y=richness, colour=Crypto)) +
  geom_boxplot(colour=1, fill=c("dodgerblue2","goldenrod1")) +
  geom_jitter(width=0.15, aes(fill=Crypto), color=1, pch=21, size=2) +
  labs(x= '', y= 'Species Richness', tag = "A") +
  scale_fill_manual(values=c("dodgerblue2", "goldenrod1")) +
  scale_x_discrete(labels = c("Control<br>(n=30)","Cryptosporidium<br>-Positive (n=30)"))
  ↪ +
  theme_classic() +
  theme(legend.position = "none", text = element_text(colour = "black", size=10),
  ↪ axis.text.x = element_markdown())

#Figure 1B
CP_Shannon <- ggplot(data_alphadiv, aes(x=Crypto, y=shannon, colour=Crypto)) +
  geom_boxplot(colour=1, fill=c("dodgerblue2","goldenrod1")) +
  geom_jitter(width=0.15, aes(fill=Crypto), color=1, pch=21, size=2) +
  labs(x= '', y= 'Shannon Index', tag = "B") +
  scale_fill_manual(values=c("dodgerblue2", "goldenrod1")) +
  scale_x_discrete(labels =c("Control<br>(n=30)","Cryptosporidium<br>-Positive (n=30)"))
  ↪ +
  theme_classic() +
  theme(legend.position = "none", text = element_text(colour = "black", size=10),
  ↪ axis.text.x = element_markdown())
```

```
dm <- vegdist(bracken, "bray")
ord <- pcoa(dm, correction="lingoes")
cpPCoA <- merge(by="row.names", metadata[,'Crypto', drop=FALSE], ord$vectors)
centroids <- aggregate(cbind(mean.x=Axis.1,mean.y=Axis.2)~Crypto, cpPCoA, mean) cpPCoA <-
↪  merge(by="Crypto", cpPCoA, centroids) cbPalette_Crypto <- c("dodgerblue2",
↪  "goldenrod1")
percent_explained <- 100 * ord$values$Corr_eig / sum(ord$values$Corr_eig)
pretty_pe <- format(round(percent_explained[1:2], digits=1), nsmall=1)
labs <- c(glue("PC1 ({pretty_pe[1]}%)"), glue("PC2 ({pretty_pe[2]}%)"))

#Figure 1C
CP_PCoA = ggplot(cpPCoA, aes(Axis.1,Axis.2,color=Crypto)) +
  theme_bw() +
  geom_segment(aes(x=mean.x, y=mean.y, xend=Axis.1, yend=Axis.2)) +
  geom_point(aes(fill=Crypto), color=1, pch=21,size=2) +
  geom_point(aes(x=mean.x,y=mean.y),colour = "black", fill = "yellow", shape = 21,
  ↪  size=3, show.legend=FALSE, inherit.aes = FALSE) +
  scale_fill_manual(values=cbPalette_Crypto,
  ↪  labels=c("Control<br>(n=30)","Cryptosporidium<br>-Positive (n=30)")) +
  scale_color_manual(values=cbPalette_Crypto,
  ↪  labels=c("Control<br>(n=30)","Cryptosporidium<br>-Positive (n=30)")) +
  labs(tag = "C", x=labs[1],y=labs[2]) +
  theme(text=element_text(size=10), legend.title=element_blank(),
  ↪  legend.text=element_markdown(size=8), legend.position = "top")

#Figure 1D
ST_Richness <- ggplot(data_alphadiv, aes(x=SwabTime, y=richness, colour=SwabTime)) +
  geom_boxplot(colour=1, fill=c("mediumpurple1","darkorange")) +
  geom_jitter(width=0.15, aes(fill=SwabTime), color=1, pch=21, size=2) +
  labs(x= '', y= 'Species Richness', tag = "D") +
  scale_fill_manual(values=c("mediumpurple1","darkorange")) +
  scale_x_discrete(labels=c("Day 1-3<br>(n=20)","Day 4-7<br>(n=40)")) +
  theme_classic() + theme(legend.position = "none", text = element_text(colour = "black",
  ↪  size=10), axis.text.x = element_markdown())

#Figure 1E
ST_Shannon <- ggplot(data_alphadiv, aes(x=SwabTime, y=shannon, colour=SwabTime)) +
  geom_boxplot(colour=1, fill=c("mediumpurple1","darkorange")) +
  geom_jitter(width=0.15, aes(fill=SwabTime), color=1, pch=21, size=2) +
  labs(x= '', y= 'Shannon Index', tag = "E") +
  scale_fill_manual(values=c("mediumpurple1","darkorange")) +
  scale_x_discrete(labels=c("Day 1-3<br>(n=20)","Day 4-7<br>(n=40)")) +
  theme_classic() +
  theme(legend.position = "none", text = element_text(colour = "black", size=10),
  ↪  axis.text.x = element_markdown())

#Figure 1F
stPCoA <- merge(by="row.names", metadata[,'SwabTime', drop=FALSE], ord$vectors) centroids
↪  <- aggregate(cbind(mean.x=Axis.1,mean.y=Axis.2)~SwabTime, stPCoA, mean)
stPCoA <- merge(by="SwabTime", stPCoA, centroids)
cbPalette_SwabTime <- c("mediumpurple1","darkorange")
ST_PCoA = ggplot(stPCoA, aes(Axis.1,Axis.2,color=SwabTime)) +
  theme_bw() +
```

```
  geom_segment(aes(x=mean.x, y=mean.y, xend=Axis.1, yend=Axis.2)) +
  geom_point(aes(fill=SwabTime), color=1, pch=21, size=2) +
  geom_point(aes(x=mean.x,y=mean.y), colour = "black", fill = "yellow", shape = 21, size=3, show.legend=FALSE, inherit.aes = FALSE) +
  scale_fill_manual(values=cbPalette_SwabTime, labels=c("Day 1-3<br>(n=20)","Day 4-7<br>(n=40)")) +
  scale_color_manual(values=cbPalette_SwabTime, labels=c("Day 1-3<br>(n=20)","Day 4-7<br>(n=40)")) +
  labs(tag = "F", x=labs[1],y=labs[2]) +
  theme(text=element_text(size=10), legend.title = element_blank(), legend.text=element_markdown(size=8), legend.position = "top")

#Statistical Comparisons
set.seed(123)

#Check normality shapiro.test(data_alphadiv$shannon)
shapiro.test(data_alphadiv$richness)

#Figure 1A
CPR = CP_Richness +
  stat_compare_means(method="t.test", label.x=0.75, label.y=80, size=3) +
  stat_compare_means(comparisons = list(c("Control (n=30)","Cryptosporidium-Positive (n=30)")), label="p.signif", method="t.test", size=3) +
  coord_cartesian(clip = "off")

#Figure 1B
CPS = CP_Shannon +
  stat_compare_means(method="wilcox.test", label.x=0.75, label.y=3.25, size=3) +
  stat_compare_means(comparisons = list(c("Control (n=30)","Cryptosporidium-Positive (n=30)")), label="p.signif", method="wilcox.test", size=3) +
  coord_cartesian(clip = "off")

#Figure 1D
STR = ST_Richness +
  stat_compare_means(method="t.test", label.x=0.75, label.y=80, size=3) +
  stat_compare_means(comparisons = list(c("Day 1-3 (n=20)","Day 4-7 (n=40)")), label="p.signif", method="t.test", size=3) +
  coord_cartesian(clip = "off")

#Figure 1E
STS = ST_Shannon +
  stat_compare_means(method="wilcox.test", label.x=0.75, label.y=3.25, size=3) +
  stat_compare_means(comparisons = list(c("Day 1-3 (n=20)","Day 4-7 (n=40)")), label="p.signif", method="wilcox.test", size=3) +
  coord_cartesian(clip = "off")

#PERMANOVA for beta diversity
metabrack <- cbind(metadata, bracken)
brack.matrix <- as.matrix(metabrack[,12:310])
brack_mat <- sqrt(brack.matrix)
brack.dist <- vegdist(brack_mat, method='bray')
ST_brack.div <- adonis2(brack.dist ~ SwabTime, data=metabrack, method="bray")
CP_brack.div<-adonis2(brack.dist ~ Crypto, data=metabrack, method="bray")
```

```
#Figure 1C
CPB = CP_PCoA +
  annotate("text", x = 0.35, y = 0.25, label = "PERMANOVA, p = 0.211", size=3) +
  annotate("text", x = 0.35, y = 0.3, label = "ns", size=3) +
  coord_cartesian(clip = "off")

#Figure 1F
STB = ST_PCoA +
  annotate("text", x = 0.35, y = 0.25, label = "PERMANOVA, p = 0.001", size=3) +
  annotate("text", x = 0.35, y = 0.3, label = "***", size=3) +
  coord_cartesian(clip ="off")

#Figure 1
ab = ggarrange(CPR, STR, CPS, STS, CPB, STB, ncol = 2, nrow = 3)

#Interaction between disease status and sampling day groups
data_alphadiv$CryptoTime <- factor(data_alphadiv$CryptoTime, levels = c("Control Day 1-3
↪  (n=7)","Cryptosporidium-Positive Day 1-3 (n=13)","Control Day 4-7
↪  (n=23)","Cryptosporidium-Positive Day 4-7 (n=17)"))

#Figure S4A
CT_Richness <- ggplot(data_alphadiv, aes(x=CryptoTime, y=richness, colour=CryptoTime)) +
  geom_boxplot(colour=1, fill=c("dodgerblue2","goldenrod1","mediumpurple1","darkorange"))
   ↪  +
  geom_jitter(width=0.15, aes(fill=CryptoTime), color=1, pch=21, size=3) +
  labs(x= '', y= 'Species Richness', tag = "A") +
   ↪  scale_fill_manual(values=c("dodgerblue2",
   ↪  "goldenrod1","mediumpurple1","darkorange")) +
  scale_x_discrete(labels = c("Control<br>Day
   ↪  1-3<br>(n=7)","Cryptosporidium<br>-Positive<br>Day 1-3<br>(n=13)","Control<br>Day
   ↪  4-7<br>(n=23)","Cryptosporidium<br>-Positive<br>Day 4-7<br>(n=17)")) +
  theme_classic() +
  theme(axis.text.x = element_markdown(colour = "black"), axis.text.y =
   ↪  element_text(colour = "black"), legend.position = "none")

CTR = CT_Richness +
  stat_compare_means(method="anova", label.y=85) +
  stat_compare_means(comparisons = list( c("Control Day 1-3 (n=7)",
   ↪  "Cryptosporidium-Positive Day 1-3 (n=13)"), c("Control Day 4-7 (n=23)",
   ↪  "Cryptosporidium-Positive Day 4-7 (n=17)")), label="p.format", method="t.test",
   ↪  size=4, label.y=71) +
  coord_cartesian(clip = "off")

#Figure S4B
CT_Shannon <- ggplot(data_alphadiv, aes(x=CryptoTime, y=shannon, colour=CryptoTime)) +
  geom_boxplot(colour=1, fill=c("dodgerblue2","goldenrod1","mediumpurple1","darkorange"))
   ↪  +
  geom_jitter(width=0.15, aes(fill=CryptoTime), color=1, pch=21, size=3) +
  labs(x= '', y= 'Shannon Index', tag = "B") +
  scale_fill_manual(values=c("dodgerblue2", "goldenrod1","mediumpurple1","darkorange")) +
  scale_x_discrete(labels = c("Control<br>Day
   ↪  1-3<br>(n=7)","Cryptosporidium-Positive<br>Day 1-3<br>(n=13)","Control<br>Day
   ↪  4-7<br>(n=23)","Cryptosporidium-Positive<br>Day 4-7<br>(n=17)")) +
```

```
  theme_classic() +
  theme(legend.position = "none", axis.text.x = element_markdown(colour = "black"),
  ↪  axis.text.y = element_text(colour = "black"))

CTS = CT_Shannon +
  stat_compare_means(method="kruskal.test", label.y=3.2) +
  stat_compare_means(comparisons=list( c("Control Day 1-3 (n=7)",
  ↪  "Cryptosporidium-Positive Day 1-3 (n=13)"), c("Control Day 4-7 (n=23)",
  ↪  "Cryptosporidium-Positive Day 4-7 (n=17)")), label="p.format",method="wilcox.test",
  ↪  size=4, label.y=2.8) +
  coord_cartesian(clip = "off")

#Figure S4C
dm <- vegdist(bracken, "bray")
ord <- pcoa(dm, correction="cailliez")
cpPCoA <- merge(by="row.names", metadata[,'CryptoTime', drop=FALSE], ord$vectors)
centroids <- aggregate(cbind(mean.x=Axis.1,mean.y=Axis.2)~CryptoTime, cpPCoA, mean)
↪  cpPCoA <- merge(by="CryptoTime", cpPCoA, centroids)
cbPalette_CryptoTime <- c("dodgerblue2", "goldenrod1","darkorange","mediumpurple1")

CT_PCoA = ggplot(cpPCoA, aes(Axis.1,Axis.2,color=CryptoTime)) +
  geom_segment(aes(x=mean.x, y=mean.y, xend=Axis.1, yend=Axis.2)) +
  geom_point(aes(fill=CryptoTime), color=1, pch=21,size=3)+
  geom_point(aes(x=mean.x,y=mean.y),colour = "black", fill = "yellow", shape = 21,
  ↪  size=3, show.legend=FALSE, inherit.aes = FALSE) +
  scale_fill_manual(values=cbPalette_CryptoTime, labels = c("Control Day 1-3
  ↪  (n=7)","Control Day 4-7 (n=23)","Cryptosporidium-Positive Day 1-3
  ↪  (n=13)","Cryptosporidium-Positive Day 4-7 (n=17)")) +
  scale_color_manual(values=cbPalette_CryptoTime, labels = c("Control Day 1-3
  ↪  (n=7)","Control Day 4-7 (n=23)","Cryptosporidium-Positive Day 1-3
  ↪  (n=13)","Cryptosporidium-Positive Day 4-7 (n=17)")) +
  labs(tag = "C", x=labs[1],y=labs[2]) +
  theme_bw() +
  theme(legend.title=element_blank(), legend.text = element_markdown(), legend.position =
  ↪  "top", axis.text=element_text(size=12), axis.title=element_text(size=12),
  ↪  legend.position = 'top') + guides(fill=guide_legend(ncol=2))

CT_brack.div<-adonis2(brack.dist ~ CryptoTime, data=metabrack, method="bray")
↪  CT_brack.div
library(pairwiseAdonis)
set.seed(123)
pairwise.adonis2(brack.dist ~ CryptoTime, data = metabrack, factors=metabrack$CryptoTime,
↪  sim_method = "bray", p_adjust_m = "bonferroni", reduce = NULL)

CTB = CT_PCoA +
  annotate("text", x = -0.25, y = -0.19, label = "Pairwise PERMANOVA, p = 0.824", size=3)
  ↪  +
  annotate("text", x = 0.35, y = 0.15, label = "Pairwise PERMANOVA, p = 0.445", size=3) +
  coord_cartesian(clip = "off")

#Figure S4
c = ggarrange(CTR, CTS, CTB, ncol = 1, nrow = 3)
```

Relative Taxa Abundance Bar Charts

```
library(tidyverse)
library(vegan)
library(RColorBrewer)
library(egg)
library(ggtext)
library(markdown)

#Phyla Relative Abundance Stacked Barcharts
bracken_phy <- read.delim(file = 'P.percent.txt', stringsAsFactors = FALSE)
bracken_phy <- t(bracken_phy)
colnames(bracken_phy) <- bracken_phy[1,]
bracken_phy <- bracken_phy[-1, ]
brack_phy<- as.data.frame(bracken_phy)
brack_phy <- rownames_to_column(brack_phy, var = "taxa")
metadata <- read.delim (file = 'calflabelmetadata.txt')
meta_phy<- as.data.frame(metadata)
brack_phy_gg1 <- brack_phy %>% gather(key="ID",value="rel_abun",-taxa)
brack_phy_gg1 <- left_join(brack_phy_gg1,meta_phy,by="ID")
brack_phy_gg1 <- as_tibble(brack_phy_gg1)
brack_phy_gg1 <- brack_phy_gg1 %>% mutate(rel_abun = as.double(brack_phy_gg1$rel_abun))

taxon_pool <- brack_phy_gg1 %>%
  group_by(taxa) %>%
  summarize(pool = max(rel_abun) < 17,
            mean = mean(rel_abun),
            .groups="drop")

brack_mut_phy <- inner_join(brack_phy_gg1, taxon_pool, by="taxa") %>%
  mutate(taxa = if_else(pool, "Other", taxa)) %>%
  group_by(Crypto, CalfLabel, taxa) %>%
  summarize(rel_abun = sum(rel_abun),
            mean = min(mean),
            .groups="drop") %>%
  mutate(taxa = factor(taxa),
         taxa = fct_reorder(taxa, mean, .desc=TRUE),
         taxa = fct_shift(taxa, n=1))

new_labels <- c("Control (n=30)"="Control (n=30)","Cryptosporidium-Positive
↪ (n=30)"="*Cryptosporidium*-Positive (n=30)")

#Figure S5A
phyla_per_sample <- ggplot(brack_mut_phy, aes(x=CalfLabel, y=rel_abun)) +
  theme_bw() +
  geom_bar(stat="identity",position="fill", aes(fill=taxa), width=1) +
  facet_grid(~Crypto, scales="free_x", space = "free_x", labeller = labeller(Crypto =
  ↪ new_labels)) +
  xlab(" ") +
  ylab( "Relative Abundance (%)") +
  scale_y_continuous(breaks = c(0.00,0.25,0.50,0.75,1.00),
                     labels = c("0","25", "50","75","100"), expand = c(0, 0)) +
  scale_fill_manual(name=NULL, values = c(brewer.pal(8, "Set1"), "green","grey",
  ↪ "blue","magenta"), labels = c("*Bacteroidetes*",
  ↪ "*Proteobacteria*","*Actinobacteria*","*Fusobacteria*","Other","*Firmicutes*")) +
```

```
  labs(tag="A") +
  theme(axis.text.x = element_text(angle=90, hjust=1, size=5),
        legend.text = element_markdown(),
        strip.text = element_markdown())

#Figure 2A
phyla_disease_status <- ggplot(brack_mut_phy, aes(x=Crypto, y=rel_abun)) +
  geom_bar(stat="identity",position="fill", aes(fill=taxa), width=0.95) +
  theme_bw() +
  xlab(" ") +
  ylab( "Relative Abundance (%)") +
  scale_y_continuous(breaks = c(0.00,0.25,0.50,0.75,1.00),
                     labels = c("0","25", "50","75","100"), expand = c(0, 0)) +
  scale_fill_manual(name=NULL, values = c(brewer.pal(8, "Set1"), "green","grey",
  ↪  "blue","magenta"), labels = c("*Bacteroidetes*",
  ↪  "*Proteobacteria*","*Actinobacteria*","*Fusobacteria*","Other","*Firmicutes*")) +
  labs(tag="A") +
  scale_x_discrete(expand = c(0.5, 0),
  ↪  labels=c("Control<br>(n=30)","*Cryptosporidium*-<br>Positive (n=30)")) +
  theme(axis.text = element_text(angle=0, size=10, colour = "black"),
        axis.title = element_text(size=12),
        axis.text.x = element_markdown(),
        legend.position="right",
        text=element_text(size=10),
        legend.text = element_markdown())

#Genus Relative Abundance Stacked Barcharts
bracken_gen <- read.delim(file = 'G.percent.txt', stringsAsFactors = FALSE)
bracken_gen <- t(bracken_gen)
colnames(bracken_gen) <- bracken_gen[1,]
bracken_gen <- bracken_gen[-1, ]
brack_gen<- as.data.frame(bracken_gen)
brack_gen <- rownames_to_column(brack_gen, var = "taxa")
metadata <- read.delim (file = 'calflabelmetadata.txt')
meta_gen<- as.data.frame(metadata)
brack_gen_gg1 <- brack_gen %>% gather(key="ID",value="rel_abun",-taxa)
brack_gen_gg1 <- left_join(brack_gen_gg1,meta_gen,by="ID")
brack_gen_gg1 <- as_tibble(brack_gen_gg1)
brack_gen_gg1 <- brack_gen_gg1 %>% mutate(rel_abun = as.double(brack_gen_gg1$rel_abun))

taxon_pool <- brack_gen_gg1 %>%
  group_by(taxa) %>%
  summarize(pool = max(rel_abun) < 14,
            mean = mean(rel_abun),
            .groups="drop")

brack_mut_gen <- inner_join(brack_gen_gg1, taxon_pool, by="taxa") %>%
  mutate(taxa = if_else(pool, "Other", taxa)) %>%
  group_by(Crypto, CalfLabel, taxa) %>%
  summarize(rel_abun = sum(rel_abun),
            mean = min(mean),
            .groups="drop") %>%
  mutate(taxa = factor(taxa),
```

```
         taxa = fct_reorder(taxa, mean, .desc=TRUE),
         taxa = fct_shift(taxa, n=1))

new_labels <- c("Control (n=30)"="Control (n=30)","Cryptosporidium-Positive
↪  (n=30)"="*Cryptosporidium*-Positive (n=30)")

#Figure S5B
genus_per_sample <- ggplot(brack_mut_gen, aes(x=CalfLabel, y=rel_abun)) +
  geom_bar(stat="identity",position="fill", aes(fill=taxa), width=1) +
  facet_grid(.~Crypto, scales="free_x", space = "free_x", labeller = labeller(Crypto =
  ↪  new_labels)) +
  xlab(" ") +
  ylab( "Relative Abundance (%)") +
  scale_y_continuous(breaks = c(0.00,0.25,0.50,0.75,1.00),
                     labels = c("0","25", "50","75","100"), expand = c(0, 0)) +
  scale_fill_manual(name=NULL, values = c(brewer.pal(8, "Set1"), "green","grey",
  ↪  "blue","magenta"),
  ↪  labels=c("*Escherichia*","*Bifidobacterium*","*Faecalibacterium*","*Blautia*",
  ↪  "*Collinsella*","*Enterococcus*","*Clostridium*","*Fusobacterium*","*Streptococcus*"
  ↪  ,"Other","*Bacteroides*")) +
  labs(tag="B") +
  theme_bw() +
  theme(axis.text.x = element_text(angle=90, hjust=1, size=5),
        legend.text = element_markdown(),
        strip.text.x = element_markdown())

# Figure 2B
genus_disease_status <- ggplot(brack_mut_gen, aes(x=Crypto, y=rel_abun)) +
  geom_bar(stat="identity",position="fill", aes(fill=taxa), width=0.95) +
  theme_bw() +
  xlab(" ") + ylab( "Relative Abundance (%)") +
  scale_y_continuous(breaks = c(0.00,0.25,0.50,0.75,1.00),
                     labels = c("0","25", "50","75","100"), expand = c(0, 0)) +
  scale_fill_manual(name=NULL, values = c(brewer.pal(8, "Set1"), "green","grey",
  ↪  "blue","magenta"),
  ↪  labels=c("*Escherichia*","*Bifidobacterium*","*Faecalibacterium*","*Blautia*",
  ↪  "*Collinsella*","*Enterococcus*","*Clostridium*","*Fusobacterium*","*Streptococcus*"
  ↪  ,"Other","*Bacteroides*")) +
  scale_x_discrete(expand = c(0.5, 0),
  ↪  labels=c("Control<br>(n=30)","*Cryptosporidium*-<br>Positive (n=30)")) +
  labs(tag="B") +
  theme(legend.position="right",
        text=element_text(size=10),
        axis.text = element_text(angle=0, size=10, colour = "black"),
        axis.title = element_text(size=12),
        axis.text.x = element_markdown(),
        legend.text = element_markdown())

rel_abun <- grid.arrange(phyla_disease_status,genus_disease_status, ncol = 1, nrow = 2)
per_samp <- grid.arrange(phyla_per_sample,genus_per_sample, ncol = 1, nrow = 2)
```

MaAsLin2 Analysis

```
#library(Maaslin2)
#library(hilldiv)
#Bracken Species Differential Abundance
df_species_data = read.table(file = "S.percent.txt", header = TRUE, sep = "\t", row.names
↪  = 1, stringsAsFactors = FALSE)
df <- t(df_species_data)
df <- tss(df)
df <- t(df)
df_metadata = read.table(file ="metadata.txt", header = TRUE, sep = "\t", row.names = 1,
↪  stringsAsFactors = FALSE)
fit_data = Maaslin2(input_data = df,
                    input_metadata = df_metadata,
                    output ="CGR_Species_Abundance_Output",
                    fixed_effects =
                    ↪  c('Crypto','Farm','Diatrim','Synulox','Halocur','SwabDay'),
                    min_abundance = 0.0001,
                    min_prevalence = 0.1,
                    normalization = "NONE",
                    transform = "NONE",
                    analysis_method = "LM",
                    max_significance = 0.25,
                    random_effects = NULL,
                    correction = "BH",
                    standardize = TRUE,
                    cores = 1,
                    plot_heatmap = TRUE,
                    plot_scatter = TRUE,
                    heatmap_first_n = 50,
                    reference=c("Farm,f2"))

#Bracken Genus Differential Abundance
df_genus_data = read.table(file = "G.percent.txt", header = TRUE, sep = "\t", row.names =
↪  1, stringsAsFactors = FALSE)
df <- t(df_genus_data)
df <- tss(df)
df <- t(df)
df_metadata = read.table(file ="metadata.txt", header = TRUE, sep = "\t", row.names = 1,
↪  stringsAsFactors = FALSE)
fit_data = Maaslin2(input_data = df,
                    input_metadata = df_metadata,
                    output ="CGR_G.percent_Abundance_Output",
                    fixed_effects =
                    ↪  c('Crypto','Farm','Diatrim','Synulox','Halocur','SwabDay'),
                    min_abundance = 0.0001,
                    min_prevalence = 0.1,
                    normalization = "NONE",
                    transform = "NONE",
                    analysis_method = "LM",
                    max_significance = 0.25,
                    random_effects = NULL,
                    correction = "BH",
```

```
                    standardize = TRUE,
                    cores = 1,
                    plot_heatmap = TRUE,
                    plot_scatter = TRUE,
                    heatmap_first_n = 50,
                    reference=c("Farm,f2"))

#Bracken Phylum Differential Abundance
df_phyla_data = read.table(file = "P.percent.txt", header = TRUE, sep = "\t", row.names =
↪  1, stringsAsFactors = FALSE)
df <- t(df_phyla_data)
df <- tss(df)
df <- t(df)
df_metadata = read.table(file ="metadata.txt", header = TRUE, sep = "\t", row.names = 1,
↪  stringsAsFactors = FALSE)
fit_data = Maaslin2(input_data = df,
                    input_metadata = df_metadata,
                    output ="CGR_P.percent_Abundance_Output",
                    fixed_effects =
                    ↪  c('Crypto','Farm','Diatrim','Synulox','Halocur','SwabDay'),
                    min_abundance = 0.0001,
                    min_prevalence = 0.1,
                    normalization = "NONE",
                    transform = "NONE",
                    analysis_method = "LM",
                    max_significance = 0.25,
                    random_effects = NULL,
                    correction = "BH",
                    standardize = TRUE,
                    cores = 1,
                    plot_heatmap = TRUE,
                    plot_scatter = TRUE,
                    heatmap_first_n = 50,
                    reference=c("Farm,f2"))

#Metaphlan Species Differential Abundance
df_metaphlan_species_data = read.table(file = "metaphlan_species.txt", header = TRUE, sep
↪  = "\t", row.names = 1, stringsAsFactors = FALSE)
df <- tss(df_metaphlan_species_data)
df_metaphlan_species_data <- t(df)
df_metadata = read.table(file ="metadata.txt", header = TRUE, sep = "\t", row.names = 1,
↪  stringsAsFactors = FALSE)
fit_data = Maaslin2(input_data = df_metaphlan_species_data,
                    input_metadata = df_metadata,
                    output ="CGR_Metaphlan_Species_Abundance_Output",
                    fixed_effects =
                    ↪  c('Crypto','Farm','Diatrim','Synulox','Halocur','SwabDay'),
                    min_abundance = 0.0001,
                    min_prevalence = 0.1,
                    normalization = "NONE",
                    transform = "NONE",
                    analysis_method = "LM",
                    max_significance = 0.25,
```

```
                    random_effects = NULL,
                    correction = "BH",
                    standardize = TRUE,
                    cores = 1,
                    plot_heatmap = TRUE,
                    plot_scatter = TRUE,
                    heatmap_first_n = 50,
                    reference=c("Farm,f2"))

#HUMAnN Pathway Abundance Differential Abundance
df_path_abund_data = read.table(file = "path_abund_destrat.txt", header = TRUE, sep =
↪  "\t", row.names = 1, stringsAsFactors = FALSE)
df <- t(df_path_abund_data)
df <- tss(df)
df_path_abund_data <- t(df)
df_metadata = read.table(file ="metadata.txt", header = TRUE, sep = "\t", row.names = 1,
↪  stringsAsFactors = FALSE)
fit_data = Maaslin2(input_data = df_path_abund_data,
                    input_metadata = df_metadata,
                    output ="CGR_Path_Abund_Output",
                    fixed_effects =
                    ↪  c('Crypto','Farm','Diatrim','Synulox','Halocur','SwabDay'),
                    min_abundance = 0.0001,
                    min_prevalence = 0.1,
                    normalization = "NONE",
                    transform = "NONE",
                    analysis_method = "LM",
                    max_significance = 0.25,
                    correction = "BH",
                    standardize = TRUE,
                    cores = 1,
                    plot_heatmap = TRUE,
                    plot_scatter = TRUE,
                    heatmap_first_n = 50,
                    reference=c("Farm,f2"))

#HUMAnN Biological Processes Differential Abundance
df_bioproc_data = read.table(file = "bio_process_destrat.txt", header = TRUE, sep = "\t",
↪  row.names = 1, stringsAsFactors = FALSE)
df <- t(df_bioproc_data)
df <- tss(df)
df_bioproc_data <- t(df)
df_metadata = read.table(file ="metadata.txt", header = TRUE, sep = "\t", row.names = 1,
↪  stringsAsFactors = FALSE)
fit_data = Maaslin2(input_data = df_bioproc_data,
                    input_metadata = df_metadata,
                    output ="CGR_Bio_Proc_Destrat_Output",
                    fixed_effects =
                    ↪  c('Crypto','Farm','Diatrim','Synulox','Halocur','SwabDay'),
                    min_abundance = 0.0001,
                    min_prevalence = 0.1,
                    normalization = "NONE",
                    transform = "NONE",
```

```
                    analysis_method = "LM",
                    max_significance = 0.25,
                    correction = "BH",
                    standardize = TRUE,
                    cores = 1,
                    plot_heatmap = TRUE,
                    plot_scatter = TRUE,
                    heatmap_first_n = 50,
                    reference=c("Farm,f2"))

#HUMAnN Cellular Components Differential Abundance
df_cellcomp_data = read.table(file = "cell_comp_destrat.txt", header = TRUE, sep = "\t",
↪  row.names = 1, stringsAsFactors = FALSE)
df <- t(df_cellcomp_data)
df <- tss(df)
df_cellcomp_data <- t(df)
df_metadata = read.table(file ="metadata.txt", header = TRUE, sep = "\t", row.names = 1,
↪  stringsAsFactors = FALSE)
fit_data = Maaslin2(input_data = df_cellcomp_data,
                    input_metadata = df_metadata,
                    output ="CGR_Cell_Comp_Destrat_Output",
                    fixed_effects =
                    ↪  c('Crypto','Farm','Diatrim','Synulox','Halocur','SwabDay'),
                    min_abundance = 0.0001,
                    min_prevalence = 0.1,
                    normalization = "NONE",
                    transform = "NONE",
                    analysis_method = "LM",
                    max_significance = 0.25,
                    correction = "BH",
                    standardize = TRUE,
                    cores = 1,
                    plot_heatmap = TRUE,
                    plot_scatter = TRUE,
                    heatmap_first_n = 50,
                    reference=c("Farm,f2"))

#HUMAnN Molecular Function Differential Abundance
df_molfunc_data = read.table(file = "mol_func_destrat.txt", header = TRUE, sep = "\t",
↪  row.names = 1, stringsAsFactors = FALSE)
df <- t(df_bioproc_data)
df <- tss(df)
df_molfunc_data <- t(df)
df_metadata = read.table(file ="metadata.txt", header = TRUE, sep = "\t", row.names = 1,
↪  stringsAsFactors = FALSE)
fit_data = Maaslin2(input_data = df_molfunc_data,
                    input_metadata = df_metadata,
                    output ="CGR_Mol_Func_Destrat_Output",
                    fixed_effects =
                    ↪  c('Crypto','Farm','Diatrim','Synulox','Halocur','SwabDay'),
                    min_abundance = 0.0001,
                    min_prevalence = 0.1,
                    normalization = "NONE",
```

```
                     transform = "NONE",
                     analysis_method = "LM",
                     max_significance = 0.25,
                     correction = "BH",
                     standardize = TRUE,
                     cores = 1,
                     plot_heatmap = TRUE,
                     plot_scatter = TRUE,
                     heatmap_first_n = 50,
                     reference=c("Farm,f2"))
```
